# Supplementary material for: Comparing the effects of interactive and conventional video education on activation, treatment adherence, and weight changes in dialysis patients: A randomized clinical trial protocol
Source: PLoS One. 2025 Oct 15;20(10):e0334498. doi: 10.1371/journal.pone.0334498 (PMC12527215; doi:10.1371/journal.pone.0334498)
Supplement: S2 File — (DOCX) [file pone.0334498.s002.docx]

# Examining and Comparing the Effects of Interactive Video-based Educational Method and Conventional Video Educational Method on Activation, Treatment Adherence and Weight Changes in Dialysis Patients: A Randomized Clinical Trial

In the Name of God

Islamic Republic of Iran

Ministry of Health and Medical Education

Shahid Beheshti University of Medical Sciences

Research Deputy

**Research Project Information**

**Name and Surname of Principal Investigator(s):** Dr. Neda Sanaei, Sogand Sarmadi ^1^

## 1. Project Proposer's Specifications

### 1-1. Name and Surname of Principal Investigator(s)

Dr. Neda Sanaei, Sogand Sarmadi

Address: Workplace:

Phone:

Home: Tehran

Phone:

Fax: Email:

### 1-2. Executing Unit

Executing Unit: Shahid Beheshti School of Nursing and Midwifery

Faculty: Shahid Beheshti School of Nursing and Midwifery Department: Medical-Surgical Nursing

Execution Location: Hospitals affiliated with Shahid Beheshti University of Medical Sciences

Duration of Execution (Months): 1 year

Other Collaborating Organizations:

### 1-3. Principal Collaborators' Specifications

| Row | Name and Surname | Occupation/Field | Academic Rank | Type of Collaboration in the Project | Signature |
| --- | --- | --- | --- | --- | --- |
| 1 | Sogand Sarmadi | Master of Nursing | Student | Investigator |  |
| 2 | Akbar Zare Kaseb | Master of Nursing | Student | Collaborator |  |
| 3 | Neda Sanaei | PhD in Medical-Surgical | Assistant Professor | Principal Investigator |  |
| 4 |  |  |  |  |  |

### 1-4. Requested Budget for the Entire Project

(Rials):

*Is the project derived from a thesis? No

## 2-1. Project Title

Persian: تاثیر و مقایسه روش آموزشی مبتنی بر ویدیوی تعاملی و روش آموزشی ویدیوی معمولی بر فعال‌سازی، تبعیت درمانی و تغییرات وزنی در بیماران دیالیزی:‌ یک کارآزمایی بالینی تصادفی سازی شده

English: Examining and Comparing the Effects of Interactive Video-based Educational Method and Conventional Video Educational Method on Activation, Treatment Adherence and Weight Changes in Dialysis Patients: A Randomized Clinical Trial

Principal Investigator(s): Dr. Neda Sanaei

## 2-2. Project Type

Applied (Checked)

Basic (Unchecked)

Basic-Applied (Unchecked)

## 2-3. Project Summary

### Background, Importance, and Necessity of the Project

Chronic Kidney Disease is one of the serious public health problems, especially in the elderly, associated with a gradual decline in kidney function, fluid homeostasis imbalance, and the need for dialysis in advanced stages. Hemodialysis is the most common treatment method in Iran, but low patient adherence to dietary and fluid restrictions, measured by interdialytic weight gain, can increase the risk of mortality, hospitalization, and reduced quality of life. "Patient activation" has been introduced as a key factor in improving adherence and self-management. Education, especially video-based, plays an effective role in enhancing awareness and treatment adherence, but the lack of interactive approaches in educating dialysis patients remains a research gap.

### Objective

Examining and Comparing the Effects of Interactive Video-based Educational Method and Conventional Video Educational Method on Activation, Treatment Adherence and Weight Changes in Dialysis Patients.

### Method

This research is a randomized controlled trial with three parallel groups, conducted on hemodialysis patients in selected hospitals affiliated with Shahid Beheshti University of Medical Sciences. Participants will be enrolled based on specific criteria and divided into three groups: interactive video education (A), conventional video education (B), and control (C). First, educational content will be designed through a literature review and validated by experts. The intervention will consist of ten 30-minute educational sessions, implemented interactively in Group A and non-interactively in Group B. The control group will receive routine education. Evaluations will be conducted at three stages: immediately, one month, and three months after the intervention. Finally, to adhere to ethical principles, all content will be provided to all three groups.

### Final Disseminators of This Research's Results

Students, Nurses, Nursing Managers, and Dialysis Units

### Keywords

Interactive video-based education, Conventional video-based education, Patient activation, Patient treatment adherence, Patient weight changes, Dialysis

## 2-4. Reasons for Topic Selection

Interest in research in the dialysis unit.

## 2-5. Problem Statement

Chronic Kidney Disease (CKD) is recognized as a major global health risk factor, with a higher prevalence in individuals over 60 years old.^1^ CKD refers to a condition of kidney impairment where more than half of the normal kidney function is lost and its definition includes evidence of kidney damage or an estimated eGFR less than 60 mL/min per 1.73 m² for at least three months . Patients with CKD are classified into five stages (1 to 5) based on their eGFR . The middle to late stages (3 to 5) of this disease significantly impact daily activities, nutritional status, fluid and electrolyte balance, and overall health, and can lead to uremic syndrome and, if untreated, to death. End-stage CKD or ESRD refers to irreversible kidney function loss requiring continuous dialysis or kidney transplantation for survival.

In Iran, based on 2016 data, over 55,000 CKD patients were reported, of whom 27,500 were undergoing hemodialysis (HD) and 1,600 were on peritoneal dialysis (PD).^1^ Subsequent studies indicate that the number of CKD patients in Iran increases by approximately 15% annually and about 27.5% has been reported. Currently, hemodialysis is the most common treatment method for ESRD in the country, but HD patients face challenges such as the necessity of strict adherence to dietary regimens to prevent cardiovascular complications. Successful implementation of a hemodialysis program requires adherence to four key components: diet, medication intake, fluid restriction, and regular attendance at dialysis sessions.^1^ Treatment adherence is defined as the patient's active participation in the treatment process, following recommendations, and receiving care services, which is manifested in their behavior . Numerous studies indicate that hemodialysis patients have poor treatment adherence, which can lead to accelerated disease progression and an increased need for emergency hospitalization [11-13].

To measure dialysis adherence, biochemical and physiological measurements such as Kt/V and interdialytic weight gain (IDWG) are used [10, 14-16]. To increase adherence and self-management in patients with chronic diseases, the concept of "patient activation" was introduced, which uses the PAM tool to measure the patient's knowledge, skills, and confidence in managing their own health . Higher activation is associated with more favorable treatment outcomes, reduced unnecessary emergency visits, and lower hospital readmission rates . Patient activation is a modifiable variable influenced by factors such as age, gender, socioeconomic status, CKD stage, disease duration, and comorbidities . This concept is defined at four levels: Level 1, where the patient feels overwhelmed and unmotivated by the disease; Level 2, where they have begun self-care but lack sufficient knowledge and confidence; Level 3, where they take action to maintain and improve health but lack the necessary skills and belief; and Level 4, where the patient actively manages their disease with sufficient knowledge and skills . Studies show that patient activation level is directly related to adherence to dietary and fluid restrictions, and low activation can lead to non-adherence to diet and increased interdialytic weight gain .

Interdialytic weight gain (IDWG), which refers to the difference in a patient's weight at the end of one dialysis session and the beginning of the next, is considered a valid indicator of fluid control and, indirectly, patient adherence to fluid intake restrictions . Although international guidelines recommend keeping IDWG below 4–4.5% of dry weight, many patients do not adhere to this limit and experience interdialytic weight gain . Numerous studies have shown that excessive IDWG is associated with an increased risk of mortality and cardiovascular mortality, including left ventricular hypertrophy and adverse cardiovascular and cerebrovascular events [24-26]. Furthermore, excessive weight gain necessitates more frequent dialysis sessions, which significantly reduces quality of life and drastically increases healthcare costs . Major barriers to adhering to dietary and fluid restrictions include insufficient understanding of body fluid status, low motivation, and underlying disease conditions that affect residual kidney function and fluid removal rates .

Education is one of the important pillars of nursing, provided through various methods . One such method is conventional video-based education, where the patient watches a pre-recorded video and receives topical information unilaterally . Studies have shown that these videos can increase patients' knowledge about disease-related care and reduce pre-treatment anxiety . Another method is interactive video-based education, where in addition to watching the film, the patient interacts with the content; by answering interactive questions, participating in virtual simulations, and receiving immediate feedback, deeper learning and motivation for adhering to treatment recommendations increase .

Despite initial evidence regarding the positive impact of passive video education on the knowledge of dialysis patients, the lack of interactive methods with the possibility of active participation and rapid feedback remains a research gap. On the other hand, the improvement of self-efficacy and treatment adherence with interactive tools in other chronic diseases indicates the high potential of this approach, but its systematic and comparative investigation in the dialysis population has not been conducted. Given the importance of interdialytic weight control and the direct impact of patient activation on adherence to fluid and medication regimens, designing a randomized clinical trial to compare the effectiveness of interactive video education versus passive video methods on activation, treatment adherence, and weight changes can lead to more effective solutions and improve the quality of care for these patients.

## 2-6. Operational Definitions of Key Concepts

### Theoretical Definition

- **Interactive Video-based Education:** In this model, in addition to watching the film, the patient interacts with the content during the education; by answering interactive questions, participating in virtual simulations, and receiving immediate feedback, the learner engages in participation .
- **Conventional Video-based Education:** In this educational approach, the patient merely watches a pre-recorded video that unilaterally conveys information about the intended topic and its aspects .
- **Dialysis:** Dialysis is a medical treatment for individuals with kidney failure. Dialysis removes waste products and excess fluids from the blood and balances the minerals needed for basic bodily functions, and it has various types (hemodialysis, peritoneal dialysis, etc.) .
- **Patient Activation Measure (PAM):** To improve adherence and self-management in chronic patients, the concept of Patient Activation has been developed using the PAM tool; this tool measures the patient's knowledge, skills, and confidence in managing their own health .
- **Patient Treatment Adherence:** Adherence to treatment means active participation in the treatment process, following recommendations, and receiving healthcare services, which is manifested in the patient's behavior .
- **Weight Changes Patients:** Interdialytic weight gain (IDWG), which refers to the difference in a patient's weight at the end of one dialysis session and the beginning of the next, is considered a valid indicator of fluid control and, indirectly, patient adherence to fluid intake restrictions .

### Operational Definition

- **Interactive Video-based Education:** In this study, interactive video-based education refers to the education provided to dialysis patients as detailed in the research methodology section.
- **Conventional Video-based Education:** In this study, conventional video-based education refers to the education provided to dialysis patients as detailed in the research methodology section.
- **Dialysis:** In this study, dialysis refers to all dialysis methods performed in the dialysis unit.
- **Patient Activation Measure (PAM):** In this study, patient activation refers to the average score obtained from the Patient Activation Measure (PAM) questionnaire developed by Hibbard et al.
- **Patient Treatment Adherence:** In this study, patient treatment adherence refers to the average score obtained from the End-Stage Renal Disease Adherence Questionnaire (ESRD-AQ).
- **Patient Weight Changes:** In this study, interdialytic weight gain (IDWG) will be measured by assessing weight before connecting to the machine and immediately after the end of each hemodialysis session, adhering to a set of standards; all weighings will be performed using a calibrated medical digital scale (with monthly calibration), patients will always be weighed wearing a standard light dialysis gown, and before that, they will empty their bladder and refrain from consuming food or drink. Additionally, all personal items (shoes, bags, jewelry) will be removed before weighing, and trained personnel or the researcher will accurately record all steps at fixed times (before the start and after the end of dialysis) to ensure data consistency and consistent comparison of weight changes throughout the treatment period.

## 2-7. Literature Review

### Lightfoot et al. (2024)

Lightfoot et al. in 2024 designed a digital health intervention titled "My Kidneys & Me" (MK&M) and investigated its impact in a randomized trial named SMILE-K on patients with Chronic Kidney Disease (CKD) stages 3 and 4. This intervention was designed to provide specialized education on health and lifestyle to patients with Chronic Kidney Disease (CKD). This study included 420 adult participants with CKD stages 3 and 4 who were randomly assigned at a 2:1 ratio to either an intervention group (n=280) or a control group (n=140). Outcomes, including "Patient Activation Measure" (PAM-13) scores, were measured at baseline and after 20 weeks, and analyses were performed using both "Complete Cases" (CC) and "Per-Protocol" (PP) approaches. Among participants, 210 (75%) used the MK&M platform more than once. The findings showed that PAM-13 scores in the intervention group increased compared to the control group at the end of week 20 (CC: 3.1 units, 95% CI: 0.2-4.6, P=0.065; PP: 3.6 units, 95% CI: 2.0-7, P=0.041), although this increase was not statistically significant in the CC analysis. Among individuals with low activation levels at baseline, a significant inter-group difference was observed in favor of the MK&M group (CC: 6.6 units, 95% CI: 1.3 to 11.9, P=0.016; PP: 9.2 units, 95% CI: 4.0 to 14.6, P<0.001). Overall, the use of the MK&M intervention led to improved patient activation compared to standard care, although this effect was not statistically significant in the overall sample, and the greatest benefit was observed among patients with low activation levels .

### Nadri et al. (2020)

Nadri et al. in 2020 designed a special educational program for chronic hemodialysis patients and investigated its effect on their adherence to dietary and fluid restrictions. In this single-center interventional study, 50 patients were allocated to two groups of 25; the control group received no education, and the intervention group was covered by the educational program. Data was collected through a demographic characteristics questionnaire, the Dialysis Diet and Fluid Non-adherence Questionnaire (DDFQ), and the Fluid Control in Hemodialysis Patients Scale (FCHPS). The results showed that after the implementation of the educational program, the mean interdialytic weight gain (kg), ultrafiltration volume, and diastolic blood pressure in the intervention group were significantly lower than in the control group. Furthermore, scores related to the four components of the DDFQ questionnaire—including frequency and severity of non-adherence to diet and frequency and severity of non-adherence to fluid restriction—were significantly better in the intervention group than in the control group. In summary, this study showed that implementing a structured educational program can lead to significant improvements in hemodialysis patients' adherence to diet and fluid restrictions and subsequent weight changes .

### Zhianfar et al. (2020)

Zhianfar et al. in 2020 implemented a multifaceted intervention aimed at improving adherence to therapeutic regimens and quality of life in End-Stage Renal Disease (ESRD) patients. This randomized controlled trial was conducted in two hemodialysis units of Shahrvand Hospital in Sari, Mazandaran Province; 70 outpatient dialysis patients, after accounting for a 10% dropout rate, were randomly assigned to intervention and control groups. The intervention included playing relevant educational videos, holding eight group cognitive-behavioral therapy sessions, and peer support through phone calls. Data was collected using demographic questionnaires, the Beck Depression Inventory (BDI-SF), the Multidimensional Social Support Scale (MSPSS), the Patient Satisfaction with Nursing Care Quality Questionnaire (PSNCQQ), the ESRD Patient Adherence Questionnaire (ESRD-AQ), and the World Health Organization Quality of Life Scale (WHOQOL-SF) at baseline and one and three months after the intervention. Results showed a significant reduction in self-reported depression symptoms (P=0.001), increased social support (P=0.001), satisfaction with nursing care (P=0.001), quality of life (P=0.001), and reduced interdialytic weight gain (IDWG) (P=0.001) in the intervention group compared to baseline. The highest growth in ESRD-AQ scores was observed one month after the intervention began, with a mean change of 131.88 units, and all subscales of this questionnaire also showed statistically significant changes. This study demonstrated that implementing a low-cost and feasible intervention without the need for major logistical or financial resources in existing healthcare systems, especially in resource-limited settings, is possible and can provide a scientific framework for evidence-based interventions in healthcare delivery .

### Zhang et al. (2025)

Zhang et al. in 2025 conducted a systematic meta-analysis of randomized controlled trials up to April 2024, aiming to investigate the effectiveness of digital health interventions on improving treatment adherence in dialysis patients. This study reviewed 17 trials with 1438 dialysis patients, and standardized mean differences (SMD) and 95% confidence intervals for adherence indicators were calculated. The quality of evidence was assessed using the GRADE method. The results showed that digital health interventions significantly improved overall treatment adherence (SMD=1.88; 95% CI: 0.46–3.29; four studies; low certainty evidence). Specifically, large improvements were observed in medication adherence (SMD=1.45; 95% CI: 0.38–2.52; four studies, 300 patients; low certainty) and dialysis session adherence (SMD=1.88; 95% CI: 0.46–3.29; four studies, 245 patients; low certainty). Moderate improvements were also reported in dietary adherence (SMD=0.58; 95% CI: 0.25–0.91; four studies, 344 patients; moderate certainty) and fluid management (SMD=−0.36; 95% CI: −0.64 to −0.07; seven studies, 619 patients; moderate certainty). Overall, this meta-analysis demonstrated that digital health interventions effectively improve various aspects of treatment adherence in dialysis patients, highlighting their value for integration into routine clinical practice .

### Pour et al. (2020)

Pour et al. in 2020 conducted a single-blind, pre-test–post-test randomized clinical trial among 63 adult hypertensive patients in a military hospital in Tehran to evaluate the effectiveness of interactive and non-interactive education via short message service (SMS) on treatment adherence and blood pressure. In this study, all patients initially received 45-minute basic education on HTN and treatment adherence, and then interactive and non-interactive SMS groups received four weekly messages for four months; the control group only had the initial education. Treatment adherence was measured by the "Hypertensive Patients Adherence Questionnaire," and systolic and diastolic blood pressure were measured at baseline and monthly. The results showed that adherence scores in the interactive SMS group significantly increased from 81.43±9.15 to 89.67±4.47 (P=0.003), while changes in the non-interactive SMS group (83.24±7.18; P=0.15) and control group (87.86±6.62; P=0.16) were not significant, and the difference between groups after the intervention was also significant (P=0.004). Although systolic and diastolic blood pressure significantly decreased in both intervention groups (P<0.05), blood pressure changes between groups did not show a statistically significant difference across the four time measurements (P>0.05). Thus, interactive SMS-based education was evaluated as effective in improving treatment adherence, but neither SMS method significantly reduced blood pressure .

## 2-8. Research Objectives and Hypotheses

### 2-8-1. General Objective

Examining and Comparing the Effects of Interactive Video-based Educational Method and Conventional Video Educational Method on Activation, Treatment Adherence and Weight Changes in Dialysis Patients.

### 2-8-2. Specific Objectives

- To determine the effect of interactive video-based education on the activation of hemodialysis patients in selected hemodialysis units affiliated with Shahid Beheshti University of Medical Sciences.
- To determine the effect of interactive video-based education on the treatment adherence of hemodialysis patients in selected hemodialysis units affiliated with Shahid Beheshti University of Medical Sciences.
- To determine the effect of interactive video-based education on weight changes in hemodialysis patients in selected hemodialysis units affiliated with Shahid Beheshti University of Medical Sciences.
- To determine the effect of conventional video-based education on the activation of hemodialysis patients in selected hemodialysis units affiliated with Shahid Beheshti University of Medical Sciences.
- To determine the effect of conventional video-based education on the treatment adherence of hemodialysis patients in selected hemodialysis units affiliated with Shahid Beheshti University of Medical Sciences.
- To determine the effect of conventional video-based education on weight changes in hemodialysis patients in selected hemodialysis units affiliated with Shahid Beheshti University of Medical Sciences.
- To compare the effectiveness of interactive video-based education with conventional video-based education on activation, treatment adherence, and weight changes in dialysis patients.

### 2-8-3. Applied Objectives

(No specific objectives listed)

### 2-8-4. Hypotheses / Questions

- Interactive video-based education is effective on the activation of hemodialysis patients in selected hemodialysis units affiliated with Shahid Beheshti University of Medical Sciences.
- Interactive video-based education is effective on the treatment adherence of hemodialysis patients in selected hemodialysis units affiliated with Shahid Beheshti University of Medical Sciences.
- Interactive video-based education is effective on weight changes in hemodialysis patients in selected hemodialysis units affiliated with Shahid Beheshti University of Medical Sciences.
- Conventional video-based education is effective on the activation of hemodialysis patients in selected hemodialysis units affiliated with Shahid Beheshti University of Medical Sciences.
- Conventional video-based education is effective on the treatment adherence of hemodialysis patients in selected hemodialysis units affiliated with Shahid Beheshti University of Medical Sciences.
- Conventional video-based education is effective on weight changes in hemodialysis patients in selected hemodialysis units affiliated with Shahid Beheshti University of Medical Sciences.
- The effectiveness of interactive video-based education is comparable to conventional video-based education on activation, treatment adherence, and weight changes in dialysis patients.

## 2-9. Research Method and Execution

### 2-9-1. Project Design and Implementation Method

#### Research Method (Type of Study, Brief Description of Work Method and Research Tools)

This study is a randomized controlled trial with three parallel groups. The research sample will consist of hemodialysis patients in selected hemodialysis units affiliated with Shahid Beheshti University of Medical Sciences. Inclusion criteria for the study are as follows: (a) willingness to participate in the study, (b) literacy, (c) being conscious and aware of time, place, and person to answer questions, (d) no history of hearing or visual impairments, (e) no cognitive impairment, (f) having a personal mobile phone or any other device capable of running interactive video and the ability to use it, (g) not using psychoactive drugs, (h) accurate diagnosis of chronic kidney disease confirmed by a nephrologist and having a medical record in the dialysis unit, and (i) age group 18 to 65 years. Exclusion criteria for the study are as follows: (a) withdrawal from the study at any stage, (b) failure to receive and watch the provided videos, (c) patient death, and (d) transfer to a treatment center outside the centers covered by Shahid Beheshti University of Medical Sciences. After approval from the ethics committee, researchers will obtain relevant permits and begin the study.

#### Development of Educational Content

Initially, an extensive literature review will be conducted to obtain the most optimal educational content. This process will involve extracting relevant articles, books, programs, and clinical and educational guidelines. In this regard, English-language databases including PubMed, Scopus, Web of Science, Embase will be systematically searched. Among Persian-language databases, SID, Magiran, Iranmedex will also be searched. Searches will be conducted using the keywords "Patient Activation," "Treatment Adherence," "Hemodialysis," and their synonyms and alternative terms. The search period will be from the inception of each database until June 2025. Then, the initial content of the program will be developed and then evaluated. Five intensive care unit nurses will evaluate the comprehensiveness of the program by completing a checklist and identify areas needing further education, and their feedback will be incorporated into the final version of the program. After completing the educational program, its content will be validated by ten faculty members and anesthesiologists. Based on expert evaluation, supplementary materials will be produced, and necessary revisions will be made.

#### Randomization and Group Allocation

After the educational content is prepared, three hospitals will be randomly selected from among the hospitals of Shahid Beheshti University of Medical Sciences. Then, to allocate the type of intervention to the centers, three envelopes will be randomly selected from nine envelopes, each bearing the name of a hospital and one of the groups A, B, or C, to determine the type of intervention for each hospital. Participants will also be assigned to groups A (interactive video), B (conventional video), and C (control group) based on their treatment location. Then, within each hospital, patients will be selected using a random number table from among all hemodialysis patients. To ensure group homogeneity, baseline demographic data and main study variables will be collected from all three groups before the intervention.

#### Intervention Implementation

Then Group A will receive interactive video. This program will consist of ten 30-minute educational sessions. The content delivery method will include lectures and question-and-answer sessions. Group B will receive conventional video; this course will consist of ten 30-minute videos delivered over ten days. The control group (C) will only receive routine education. Immediately after the completion of the educational sessions, and one and three months thereafter, the desired outcomes will be evaluated. After the completion of evaluations, to adhere to ethical principles, all provided content will be given to all three groups.

#### Education Method for the Interactive Video Intervention Group

This educational intervention will consist of two phases, totaling six hours of implementation. Patients will participate in this study in groups of 5, guided by an experienced instructor familiar with the interactive video education system.

**Phase One – Interactive Video-based Education (10 sessions of 30 minutes):** Patients will access the system via the website https://spotplayer.ir/ and extensively watch the interactive video related to end-stage kidney disease and alternative treatments. This video will consist of ten 30-minute sessions, uploaded daily for patients. During viewing, pop-up questions will appear, which patients must answer. Different choices will lead to different educational scenarios. Also, in parts of the video, multiple-choice questions about related concepts will be provided, which patients must record their answers in the system, and the instructor will be able to view the answers in the management panel. It should be noted that all patients will have received the necessary training beforehand on how to use interactive videos, and necessary Q&A sessions will be conducted to ensure high proficiency in using interactive video.

**Phase Two – Summary and Consolidation of Education (1 hour):** After completing the ten interactive video sessions, patients will engage in group discussions and analysis of the scenarios, materials, and questions raised. The instructor will guide the discussions, summarize the results, and evaluate the overall performance of the groups. In this phase, guided by the instructor, patients will explore complex aspects of the disease and extract key information. Finally, the instructor will complement and reinforce patient analyses by providing targeted theoretical explanations.

#### Education Method for the Conventional Video Intervention Group

This educational intervention will also consist of two phases, totaling six hours of implementation. Patients will participate in this study in groups of 5, guided by an experienced instructor familiar with the interactive video education system.

**Phase One – Conventional Video-based Education (10 sessions of 30 minutes):** Patients will access the system via the website https://spotplayer.ir/ and extensively watch the videos related to end-stage kidney disease and alternative treatments. This video will consist of ten 30-minute sessions, uploaded daily for patients.

**Phase Two – Summary and Consolidation of Education (1 hour):** After completing the ten video sessions, patients will engage in group discussions and analysis of the scenarios, materials, and questions raised. The instructor will guide the discussions, summarize the results, and evaluate the overall performance of the groups. In this phase, guided by the instructor, patients will explore complex aspects of the disease and extract key information. Finally, the instructor will complement and reinforce patient analyses by providing targeted theoretical explanations.

#### Control Group

This group will receive the routine education of each center, which will include patient education by nurses, educational pamphlets, and educational follow-ups by the follow-up nurse of the unit.

### 2-9-2. Type of Study

Three-group clinical trial

### 2-9-3. Data Collection Method

Through separate questionnaires.

### 2-9-4. Tools and Methods of Data Collection

#### Demographic Information Questionnaire

This questionnaire consists of two sections:

- **Section One: Demographic Data** includes age, gender, educational status, marital status, duration of disease diagnosis, duration since the first dialysis, employment status, place of residence, and comorbidities.
- **Section Two: Clinical Data** includes daily salt intake (in grams), complications, etc., and urine output (in milliliters per day), number of hemodialysis sessions per week, water intake, which will be collected from participants' medical records before the intervention at baseline.

#### Patient Activation Measure (PAM)

The Patient Activation Measure (PAM) scale, developed by Hibbard et al., consists of 13 questions and is considered a valid and reliable tool for measuring patient activation in the field of nephrology . Responses on this scale are recorded based on a five-point Likert scale ("Strongly disagree" = 1, "Disagree" = 2, "Agree" = 3, "Strongly agree" = 4, and "Not applicable" = no score) and raw scores range from 13 to 52; lower scores indicate less activation, and higher scores indicate more activation . According to the scoring guidelines provided by Hibbard et al., the overall activation score is standardized to a 0–100 scale and categorized into four levels: Level 1 "Disengaged and overwhelmed" (score ≤ 47), Level 2 "Becoming aware of self-management tasks" (47.1–55.1), Level 3 "Taking action" (55.2–67), and Level 4 "Maintaining behaviors and further progress" (> 67.1). Previous studies have confirmed the reliability of this scale with a Cronbach's alpha coefficient of 87% . The Cronbach's alpha coefficient for the Persian version of PAM was 91% .

#### Treatment Adherence Questionnaire (ESRD-AQ)

The End-Stage Renal Disease Adherence Questionnaire (ESRD-AQ) is a self-report tool consisting of 46 questions across five sections, designed to assess patient adherence to treatment in four dimensions: hemodialysis session attendance, medication intake, fluid restriction, and dietary recommendations. The first section of this questionnaire asks for general patient information regarding ESRD status and history of kidney replacement therapy (5 questions), and the next four sections, respectively, include adherence to hemodialysis attendance (14 questions), medication intake (9 questions), fluid restriction (10 questions), and dietary recommendations (8 questions). Responses in this tool are recorded using a combination of Likert scale, multiple-choice questions, and "yes/no" format. Total final scores range from 0 to 1200, with higher scores indicating a higher level of treatment adherence . The reliability of this tool was confirmed by Rafiei et al. (2014) with a Cronbach's alpha coefficient of 0.91 and a test-retest reliability coefficient of 0.85 ; Kim et al. (2010) also reported its content validity with a Content Validity Index (CVI) of 0.99 .

#### Patient Weight Changes (IDWG)

In this study, interdialytic weight gain (IDWG) will be measured by assessing weight before connecting to the machine and immediately after the end of each hemodialysis session, adhering to a set of standards; all weighings will be performed using a calibrated medical digital scale (with monthly calibration), patients will always be weighed wearing a standard light dialysis gown, and before that, they will empty their bladder and refrain from consuming food or drink. Additionally, all personal items (shoes, bags, jewelry) will be removed before weighing, and trained personnel or the researcher will accurately record all steps at fixed times (before the start and after the end of dialysis) to ensure data consistency and consistent comparison of weight changes throughout the treatment period.

### 2-9-5. Study Population

Dialysis patients referring to the dialysis units of hospitals affiliated with Shahid Beheshti University of Medical Sciences.

### 2-9-6. Sampling Method and Sample Size Calculation

Given the absence of previous studies with close similarity in design and interventions to the current trial, the sample size calculation will be based on data obtained from a preliminary pilot study conducted by the research team. This pilot study will provide estimates of effect size and variability, which will be used to calculate the required sample size for this three-group randomized controlled trial with four measurement times. This approach ensures that the sample size has sufficient statistical power to detect clinically meaningful differences between groups, considering the specific characteristics of the study population and interventions. Based on the pilot data, the effect size will be estimated, and considering a significance level of 0.05 (two-sided) and a statistical power of 95%, the overall estimated sample size will be equally divided among the three study groups. By relying on pilot data in the absence of previous sources, the study design achieves the necessary methodological rigor while ensuring optimal resource utilization and maintaining sufficient statistical power to address the main research objectives.

### 2-9-7. Data Analysis Methods

For the analysis of quantitative data, appropriate descriptive statistics, including mean and standard deviation for symmetrically distributed data and median and interquartile range for asymmetric and skewed distributions, will be used. Qualitative data will be reported with frequencies and percentages. Pearson's correlation coefficient will be reported if variables are normally distributed, or its non-parametric equivalent, Spearman's correlation coefficient, if distributions are not normal. Generalized Linear Modeling (GLM) will be used to adjust for confounders in examining the relationship between variables. Furthermore, the relationship between the variables of interest and quantitative demographic variables will be examined using correlation coefficients, and with dichotomous qualitative demographic variables using independent t-tests, and with multi-state qualitative variables using Analysis of Variance (ANOVA), and if necessary, their non-parametric equivalents. Analyses will be performed using SPSS 26 software. Tests will be conducted at a significance level of 5% and will be two-sided.

### 2-9-9. Variables Table

| Variable Name | Variable Role | Variable Type (Quantitative: Discrete/Continuous; Qualitative: Ordinal/Nominal) | Unit of Measurement | Measurement Method |
| --- | --- | --- | --- | --- |
| Age | Background | Quantitative (Continuous) | Years | Demographic Questionnaire |
| Gender | Background | Qualitative (Nominal) | Male, Female | Demographic Questionnaire |
| Marital Status | Background | Qualitative (Nominal) | Married, Single | Demographic Questionnaire |
| Place of Residence | Background | Qualitative (Nominal) | City, Village | Demographic Questionnaire |
| Education | Background | Qualitative (Ordinal) | Below Diploma, Diploma, Bachelor's and above | Demographic Questionnaire |
| Duration of Disease Diagnosis | Background | Quantitative (Continuous) | Months | Demographic Questionnaire |
| Duration since First Dialysis | Background | Quantitative (Continuous) | Months | Demographic Questionnaire |
| Employment Status | Background | Qualitative (Nominal) | Employed, Unemployed, Retired | Demographic Questionnaire |
| Comorbidities | Background | Qualitative (Nominal) | Diabetes, Hypertension, Depression, Anemia, Heart Failure, Other | Demographic Questionnaire |
| Complications | Background | Qualitative (Nominal) | Disequilibrium Syndrome, Hypotension, Other | Medical Records |
| Number of Hemodialysis Sessions per Week | Background | Quantitative (Discrete) | Number | Medical Records |
| Water Intake | Background | Quantitative (Continuous) | Milliliters | Medical Records |
| Daily Salt Intake | Background | Quantitative (Continuous) | Grams | Medical Records |
| Urine Output | Background | Quantitative (Continuous) | Milliliters per day | Medical Records |
| Patient Weight Changes | Dependent | Quantitative (Continuous) | Calibrated Medical Digital Scale | Weight measurement before connecting to machine and immediately after end of each hemodialysis session |
| Patient Activation | Dependent | Quantitative (Continuous) | Standard Questionnaire | Patient Activation Measure Questionnaire |
| Treatment Adherence | Dependent | Quantitative (Continuous) | Standard Questionnaire | End-Stage Renal Disease Adherence Questionnaire |

## 2-10. Project Implementation Stages (Gantt Chart)

| Project Implementation Stages | Time in Months | Percentage of Work Progress |
| --- | --- | --- |
| Data Collection, Questionnaire Completion, and Software Entry | 1-5 |  |
| Data Analysis | 6-8 |  |
| Review, Conclusion, and Text Writing | 9-12 |  |

## 2-11. Ethical Considerations

The ethical considerations addressed in this research include:

Ethical Codes: Codes 1, 11, 12, 13, 14, 15, 16, 17, 19, 20, 25, 27, 28, 29, 30, 31.

1. Obtaining written permission from the esteemed Dean of the School of Nursing and Midwifery, Shahid Beheshti University of Medical Sciences.
2. Obtaining permission from the Research Deputy of the School of Nursing and the Research Deputy of Shahid Beheshti University of Medical Sciences.
3. Providing an introduction letter and obtaining permission from responsible authorities.
4. Introducing oneself to the research units and explaining the objectives and nature of the research to them, and obtaining their written informed consent.
5. Explaining to the research units about the voluntary nature of participation in the research and the freedom to withdraw from the research, as well as the confidentiality of information and no need to mention names in the demographic information form.
6. Providing a summary of the research results to the officials of the studied hospitals and all stakeholders upon request.

## 2-12. Project Limitations, Potential Systematic Errors, and Ways to Address Them

Currently, there are no specific limitations for this study.

## 2-13. Budget and Expenses

### 2-12-1. Personnel Costs (with full specifications, employment rate, and remuneration):

| Row | Type of Activity | Academic Rank | Number of Individuals | Total Work Hours for Project | Remuneration per Hour | Total |
| --- | --- | --- | --- | --- | --- | --- |
|  |  |  |  |  |  |  |
|  |  |  |  |  |  |  |
|  |  |  |  |  |  |  |
|  |  |  |  |  |  |  |
| **Total** |  |  |  |  |  |  |

### 2-12-2. Costs of Tests and Specialized Services provided by other institutions:

| Cost of Test or Specialized Service | Service Provider Center | Total Number of Times | Cost per Time | Total (Rials) |
| --- | --- | --- | --- | --- |
|  |  |  |  |  |
|  |  |  |  |  |
|  |  |  |  |  |
|  |  |  |  |  |
| **Total** |  |  |  |  |

### 2-12-3. List of Equipment and Materials to be purchased from project funds, domestically or internationally:

#### A- Non-consumable materials (Capital):

| Device Name | Country of Manufacture | Manufacturing Company | Is it available in Iran? | Iranian Seller Company | Quantity Needed | Unit Price | Total Price |
| --- | --- | --- | --- | --- | --- | --- | --- |
|  |  |  |  |  |  |  |  |
|  |  |  |  |  |  |  |  |
|  |  |  |  |  |  |  |  |
| **Total** |  |  |  |  |  |  |  |

#### B- Consumable materials:

| Material Name | Country of Manufacture | Manufacturing Company | Is it available in Iran? | Iranian Seller Company | Quantity Needed | Unit Price | Total Price |
| --- | --- | --- | --- | --- | --- | --- | --- |
|  |  |  |  |  |  |  |  |
|  |  |  |  |  |  |  |  |
|  |  |  |  |  |  |  |  |
| **Total** |  |  |  |  |  |  |  |

### 2-12-4. Travel Costs (if necessary):

| Destination | Number of Trips during Project Execution and Purpose | Type of Vehicle | Number of Individuals | Cost in Rials |
| --- | --- | --- | --- | --- |
|  |  |  |  |  |
|  |  |  |  |  |
|  |  |  |  |  |
| **Total** |  |  |  |  |

### 2-12-5. Miscellaneous Expenses:

| Item | Total Price |
| --- | --- |
| Cost of duplicating forms and questionnaires needed for the project |  |
| Cost of duplicating publications and books needed for the project |  |
| Other expenses (specify)............. |  |
| **Total** |  |

### 2-10-6. Total Project Costs (Rials):

| Item | Total (Rials) |
| --- | --- |
| Total Personnel Costs (2-10-1) |  |
| Total Costs of Tests and Specialized Services (2-10-2) |  |
| Total Costs of Non-consumable Materials (2-10-3) A |  |
| Total Costs of Consumable Materials (4-10-3) B |  |
| Total Travel Costs (2-10-4) |  |
| Total Miscellaneous Expenses (2-10-5) |  |
| **Grand Total** |  |
