# Supplementary material for: Comparing the effects of interactive and conventional video education on activation, treatment adherence, and weight changes in dialysis patients: A randomized clinical trial protocol
Source: PLoS One. 2025 Oct 15;20(10):e0334498. doi: 10.1371/journal.pone.0334498 (PMC12527215; doi:10.1371/journal.pone.0334498)
Supplement: S3 File — (DOC) [file pone.0334498.s003.doc]

# **سهبسمه تعالي**

# **
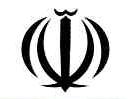
**

#

# **
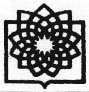
**

**جمهوري اسلامي ايران**

**وزارت بهداشت، درمان و آموزش پزشكي**

**دانشگاه علوم پزشکی شهيد بهشتی**

**معاونت پژوهشی**

**اطلاعات مربوط به پروژه تحقيقاتي**

**عنوان پروژه:**

فارسي : **تاثیر و مقایسه روش آموزشی مبتنی بر ویدیوی تعاملی و روش آموزشی ویدیوی معمولی بر فعال‌سازی، تبعیت درمانی و تغییرات وزنی در بیماران دیالیزی:‌ یک کارآزمایی بالینی تصادفی سازی‌شده** ________________________________________________________

انگليسي :

**Examining and Comparing the Effects of Interactive Video-based Educational Method and Conventional Video Educational Method on Activation, Treatment Adherence and Weight Changes in Dialysis Patients: A Randomized Clinical Trial**

**نام و نام خانوادگي مجري (مجريان):**

دکتر ندا ثنایی، سوگند سرمدی

**نشاني:** تهران بزرگراه چمران –اوين –خيابان تابناک-جنب بيمارستان طالقانی-دانشگاه علوم پزشکی شهيد بهشتی-معاونت پژوهشی **تلفن:** 22439951(021)

**مشخصات ارايه دهنده پروژه**

**1-1- نام و نام‌خانوادگي مجري (مجريان):** دکتر ندا ثنایی، سوگند سرمدی

**نشاني: محل کار: تلفن:**

**منزل: تهران تلفن:**

**نمابر: پست الكترونيك:**

**1-2- واحد اجراكننده:** دانشکده پرستاری مامایی شهید بهشتی

**دانشكده:** پرستاری مامایی شهید بهشتی **گروه:** پرستاری داخلی جراحی

**محل اجرا:** بیمارستان های زیرمجموعه دانشگاه علوم پزشکی شهید بهشتی

**مدت اجرا ( ماه ):** 1 سال

**ساير سازمان‌هاي همكار:**

**1-3- م**شخصات همكاران اصلي:

| رديف | نام و نام‌خانوادگي | شغل/رشته | رتبه علمي | نوع همكاري در طرح | امضا |
| --- | --- | --- | --- | --- | --- |
| 1  2  3  4 | سوگند سرمدی  اکبر زارع کاسب  ندا ثنایی | کارشناسی ارشد پرستاری  کارشناسی ارشد پرستاری  دکترا داخلی جراحی | دانشجو  دانشجو  استادیار | مجری  همکار  مجری اصلی |  |

**1-4- بودجه درخواستي براي كل طرح (ريال):**

***آیا طرح برگرفته از پایان نامه است؟** خیر

**2-1-** **عنوان پروژه:**

**فارسي** :

**تاثیر و مقایسه روش آموزشی مبتنی بر ویدیوی تعاملی و روش آموزشی ویدیوی معمولی بر فعال‌سازی، تبعیت درمانی و تغییرات وزنی در بیماران دیالیزی:‌ یک کارآزمایی بالینی تصادفی سازی شده**

**انگليسي** :

**Examining and Comparing the Effects of Interactive Video-based Educational Method and Conventional Video Educational Method on Activation, Treatment Adherence and Weight Changes in Dialysis Patients: A Randomized Clinical Trial**

**مجری/مجریان:** دکتر ندا ثنایی

**2-2- نوع پروژه: كاربردي◼ بنيادي❑ بنيادي ـ كاربردي❑**

**2-3- خلاصه پروژه :**

**پیشینه اهمیت وضرورت انجام پروژه:**

بیماری مزمن کلیه یکی از معضلات جدی سلامت عمومی به‌ویژه در سالمندان است که با کاهش تدریجی عملکرد کلیه، اختلال در هموستاز مایعات و نیاز به دیالیز در مراحل پیشرفته همراه می‌شود. همودیالیز شایع‌ترین روش درمان در ایران است، اما پایبندی پایین بیماران به محدودیت‌های رژیم غذایی و مایعات که با شاخص افزایش وزن بین جلسات دیالیز سنجیده می‌شود، می‌تواند خطر مرگ‌ومیر، بستری و کاهش کیفیت زندگی را افزایش دهد. «فعال‌سازی بیمار» به‌عنوان عاملی کلیدی در بهبود پایبندی و خودمدیریتی معرفی شده است. آموزش به‌ویژه به‌صورت ویدیویی، نقش مؤثری در ارتقای آگاهی و تبعیت درمانی دارد، اما کمبود رویکردهای تعاملی در آموزش بیماران دیالیزی به‌عنوان یک خلأ پژوهشی باقی مانده است.

**هدف:**

تاثیر و مقایسه روش آموزشی مبتنی بر ویدیوی تعاملی با روش آموزشی مبتنی بر ویدیوی معمولی بر فعال‌سازی، تبعیت درمانی و تغییرات وزنی در بیماران دیالیزی

**روش:**

این پژوهش یک کارآزمایی تصادفی کنترل‌شده با سه گروه موازی است که بر روی بیماران همودیالیزی در بیمارستان‌های منتخب دانشگاه علوم پزشکی شهید بهشتی انجام می‌شود. شرکت‌کنندگان بر اساس معیارهای مشخص وارد مطالعه شده و به سه گروه آموزش ویدیوی تعاملی (A)، ویدیوی معمولی (B) و کنترل (C) تقسیم می‌شوند. ابتدا محتوای آموزشی با مرور متون علمی طراحی و با نظر کارشناسان اعتبارسنجی می‌شود. مداخله شامل ده جلسه آموزشی ۳۰ دقیقه‌ای است که در گروه A به‌صورت تعاملی و در گروه B به‌صورت غیرتعاملی اجرا می‌شود. گروه کنترل آموزش روتین را دریافت می‌کند. ارزیابی‌ها در سه مرحله: بلافاصله، یک ماه و سه ماه پس از مداخله انجام خواهد شد. در پایان، برای رعایت اصول اخلاقی، تمامی محتواها در اختیار هر سه گروه قرار می‌گیرد.

**معرف کنندگان نهایی نتایج این تحقیق چه افراد یا سازمانهایی هستند**

دانشجویان، پرستاران و مدیران پرستاری و بخش های دیالیز

**واژگان کلیدی(5_3واژه):** آموزش مبتنی بر ویدیوی تعاملی، آموزش مبتنی بر ویدیوی معمولی، فعال سازی بیماران، تبعیت درمانی بیماران، تغییرات وزنی بیماران، دیالیز

2_4 دلايل انتخاب موضوع: علاقه به تحقیق در بخش دیالیز

**5- بيان مسئله Problem statement)**):

بیماری مزمن کلیه (CKD) به‌عنوان یک عامل خطر عمده سلامت در سطح جهانی شناخته می‌شود که شیوع آن در افراد بالای ۶۰ سال بالاتر است (1). CKD اصطلاحی است برای حالتی از اختلال کلیوی که طی آن بیش از نیمی از کارکرد طبیعی کلیه‌ها از بین می‌رود (2) و تعریف آن شامل وجود نشانه‌ای از آسیب کلیوی یا برآورد eGFR کمتر از ۶۰ میلی‌لیتر در دقیقه به ازای هر ۱.۷۳ متر مربع به مدت دست‌کم سه ماه است(3). بیماران مبتلا به CKD در پنج مرحله (از ۱ تا ۵) طبقه‌بندی می‌شوند که این طبقه‌بندی مبتنی بر میزان eGFR است (4). مراحل میانی تا انتهایی (۳ تا ۵) این بیماری، تأثیرات قابل‌توجهی بر فعالیت‌های روزمره، وضعیت تغذیه‌ای، تعادل آب و الکترولیت و کلیت سلامت فرد دارند و می‌توانند به سندرم اورمیک و در صورت عدم مداخله درمانی به مرگ بینجامند (5). مرحله نهایی CKD یا ESRD به کاهش برگشت‌ناپذیر عملکرد کلیه اشاره دارد که برای ادامه حیات نیازمند دیالیز مداوم یا پیوند کلیه است (6).

در ایران، بر اساس داده‌های سال ۲۰۱۶ بیش از ۵۵ هزار بیمار CKD گزارش شده که از این میان ۲۷۵۰۰ نفر تحت همودیالیز (HD) و ۱۶۰۰ نفر تحت دیالیز صفاقی (PD) قرار داشتند (1). مطالعات بعدی حاکی از این است که شمار بیماران CKD در ایران به طور سالانه حدود ۱۵٪ افزایش می‌یابد و حدود ۲۷.۵ درصد گزارش شده است (1, 7). هم‌اکنون همودیالیز رایج‌ترین روش درمان ESRD در کشور است (8) اما بیماران تحت HD با چالش‌هایی همچون ضرورت رعایت دقیق رژیم غذایی برای پیشگیری از عوارض قلبی‌عروقی مواجه‌اند (9). اجرای موفق برنامه همودیالیز مستلزم پایبندی به چهار مؤلفه کلیدی شامل رژیم غذایی، مصرف دارو، محدودیت مصرف مایعات و حضور منظم در جلسات دیالیز می‌باشد (1).

پایبندی به درمان به معنای مشارکت فعال بیمار در روند درمان، پیروی از توصیه‌ها و دریافت خدمات مراقبتی تعریف می‌شود که در رفتار وی نمود پیدا می‌کند (10). مطالعات متعدد بیانگر این است که بیماران تحت همودیالیز پایبندی ضعیفی به درمان دارند که این مسئله می‌تواند منجر به تسریع پیشرفت بیماری و افزایش نیاز به بستری اورژانسی شود (11-13). برای سنجش میزان پایبندی دیالیزی از اندازه‌گیری‌های بیوشیمیایی و فیزیولوژیک مثل Kt/V و افزایش وزن بین جلسات دیالیز (IDWG) استفاده می‌شود (10, 14-16).

در جهت افزایش پایبندی و خودمدیریتی بیماران مبتلا به بیماری‌های مزمن، مفهوم «فعال‌سازی بیمار» عرضه شد که با ابزار PAM میزان دانش، مهارت و اعتماد به نفس بیمار در مدیریت سلامت خود را اندازه‌گیری می‌کند (17). فعال‌سازی بالاتر با نتایج درمانی مطلوب‌تر، کاهش مراجعات غیرضروری به اورژانس و نرخ پایین‌تر بستری مجدد در بیمارستان همراه است (18). فعال‌سازی بیمار یک متغیر قابل تغییر است که عواملی نظیر سن، جنسیت، وضعیت اقتصادی-اجتماعی، مرحله CKD، مدت زمان بیماری و بیماری‌های همراه بر آن تأثیرگذارند (17, 19). این مفهوم در چهار سطح تعریف می‌شود: سطح ۱ که بیمار در آن احساس غرق شدن در بیماری و بی‌انگیزگی دارد، سطح ۲ که مراقبت از خود را آغاز کرده اما دانش و اعتماد کافی را کسب نکرده است، سطح ۳ که اقدام به حفظ و بهبود سلامت می‌کند اما فاقد مهارت و باور لازم است و سطح ۴ که بیمار با دانش و مهارت کافی نقش فعالی در مدیریت بیماری دارد (20). مطالعات نشان می‌دهند که سطح فعال‌سازی بیمار با میزان رعایت محدودیت‌های غذایی و مایعات ارتباط مستقیم دارد و فعال‌سازی پایین می‌تواند منجر به عدم رعایت رژیم غذایی و افزایش وزن بین جلسات دیالیز شود (21, 22).

افزایش وزن بین جلسات دیالیز، که به اختلاف وزن بیمار در پایان یک جلسه دیالیز و ابتدای جلسه‌ی بعد اشاره دارد، به‌عنوان شاخص معتبری از کنترل مایعات و به‌طور غیرمستقیم پایبندی بیمار به محدودیت‌های مصرف مایعات در نظر گرفته می‌شود (15). اگرچه دستورالعمل‌های بین‌المللی توصیه می‌کنند IDWG را زیر ۴–۴٫۵٪ وزن خشک نگه دارند، بسیاری از بیماران این حد را رعایت نکرده و با افزایش وزن بین جلسات مواجه می‌شوند (16, 23). مطالعات متعددی نشان داده‌اند که IDWG بیش از حد با افزایش خطر مرگ‌ومیری و مرگ‌ومیر قلبی‌عروقی، از جمله پرفشاری بطن چپ و حوادث قلبی و مغزی ناگوار همراه است (24-26). علاوه بر این، وزن‌گیری اضافی نیاز به جلسات دیالیز فراوان‌تر را ایجاد می‌کند که کیفیت زندگی را به‌طور قابل‌توجهی کاهش و هزینه‌های مراقبت‌های بهداشتی را به‌شدت افزایش می‌دهد (27, 28). موانع عمده در رعایت رژیم غذایی و محدودیت مایعات، از جمله شناخت ناکافی وضعیت مایعات بدن، انگیزه کم و شرایط زمینه‌ای بیماری که بر عملکرد باقی‌مانده کلیه و نرخ حذف مایعات تأثیر می‌گذارند، در این زمینه نقش دارند (28, 29).

آموزش یکی از ارکان مهم پرستاری است که به روش‌های گوناگون ارائه می‌شود (30). یکی از این روش‌ها آموزش مبتنی بر ویدیوی معمولی است که بیمار به تماشای ویدیوی از پیش ضبط‌شده می‌پردازد و اطلاعات موضوعی را به‌صورت یک‌طرفه دریافت می‌کند (31). مطالعات نشان داده‌اند که این ویدیوها می‌توانند دانش بیماران را در مورد مراقبت‌های مرتبط با بیماری افزایش و اضطراب پیش از درمان را کاهش دهند (31, 32). روش دیگر، آموزش مبتنی بر ویدیوی تعاملی است که علاوه بر تماشای فیلم، بیمار با محتوا تعامل دارد؛ با پاسخ به سؤال‌های تعاملی، شرکت در شبیه‌سازی‌های مجازی و دریافت بازخورد فوری، یادگیری عمیق‌تر و انگیزه برای رعایت توصیه‌های درمانی بالا می‌رود (33, 34).

با وجود شواهد اولیه در مورد تأثیر مثبت آموزش ویدیویی غیرفعال بر دانش بیماران دیالیزی، کمبود روش‌های تعاملی با امکان مشارکت فعال و بازخورد سریع، به‌عنوان یک خلأ تحقیقاتی باقی است. از سوی دیگر، بهبود خودکارآمدی و پایبندی درمانی با ابزارهای تعاملی در سایر بیماری‌های مزمن نشان‌دهنده پتانسیل بالای این رویکرد است، اما بررسی نظام‌مند و مقایسه‌ای آن در جمعیت دیالیزی انجام نشده است. با توجه به اهمیت کنترل وزن بین جلسات دیالیز و تأثیر مستقیم فعال‌سازی بیمار بر انطباق با رژیم مایعات و دارو، طراحی یک کارآزمایی بالینی تصادفی برای مقایسه اثربخشی آموزش ویدیویی تعاملی در برابر روش ویدیوی غیرفعال بر متغیرهای فعال‌سازی، پایبندی درمانی و تغییرات وزنی، می‌تواند به ارائه راهکارهای مؤثرتر و ارتقای کیفیت مراقبت از این بیماران منجر شود.

**2_6_تعاریف عملیاتی مفاهیم اصلی:**

تعریف نظری:

1. **Interactive Video-based Education (آموزش مبتنی بر ویدیوی تعاملی):** در این مدل، علاوه بر تماشای فیلم، بیمار در طول آموزش با محتوا تعامل می‌کند؛ با پاسخ به سؤال‌های تعاملی، شرکت در شبیه‌سازی‌های مجازی و دریافت بازخورد فوری فراگیر به مشارکت می پردازد (33, 34).
2. **Conventional Video-based Education (آموزش مبتنی بر ویدیوی معمولی):** در این رویکرد آموزشی، بیمار صرفاً به تماشای ویدیویی از پیش ضبط‌شده می‌پردازد که اطلاعات مربوط به موضوع مدنظر و جوانب آن را به صورت یک‌طرفه منتقل می‌کند (31).
3. **Dialysis (دیالیز):** دیالیز یک درمان پزشکی برای افراد مبتلا به نارسایی کلیه است. دیالیز مواد زائد و مایعات اضافی را از خون خارج کرده و مواد معدنی مورد نیاز برای عملکردهای اساسی بدن را متعادل می‌کند و انواع مختلفی(همودیالیز و دیالیز و ...) صفاقی دارد (35).
4. **Patients Activation Measure(فعال سازی بیماران):** برای بهبود پایبندی و خودمدیریتی بیماران مزمن، مفهوم فعال‌سازی بیمار (Patient Activation)با استفاده از ابزار PAM توسعه یافته است؛ این ابزار میزان دانش، مهارت و اطمینان بیمار در مدیریت سلامت خویش را می‌سنجد (17).
5. **Patients Treatment Adherence (تبعیت درمانی بیماران):** پایبندی به درمان به معنای مشارکت فعال در فرایند درمان، پیروی از توصیه‌ها و دریافت خدمات مراقبت‌های بهداشتی است که در رفتار بیمار تظاهر می‌یابد (10).
6. **Weight Changes Patients (تغییرات وزنی بیماران):** افزایش وزن بین جلسات دیالیز (IDWG)، که به اختلاف وزن بیمار در پایان یک جلسه دیالیز و ابتدای جلسه‌ی بعد اشاره دارد، به‌عنوان شاخص معتبری از کنترل مایعات و به‌طور غیرمستقیم پایبندی بیمار به محدودیت‌های مصرف مایعات در نظر گرفته می‌شود (15).

تعریف عملی:

1. **Interactive Video-based Education (آموزش مبتنی بر ویدیوی تعاملی):** در این مطالعه منظور از آموزش مبتنی بر ویدیو تعاملی آموزش ارائه شده به بیماران دیالیزی مطابق آنچه در قسمت روش اجرای تحقیق به تفضیل ارائه شده می باشد.
2. **Conventional Video-based Education (آموزش مبتنی بر ویدیوی معمولی):** در این مطالعه منظور از آموزش مبتنی بر ویدیو معمولی آموزش ارائه شده به بیماران دیالیزی مطابق آنچه در قسمت روش اجرای تحقیق به تفضیل ارائه شده می باشد.
3. **Dialysis (دیالیز):** در این مطالعه منظور از دیالیز، تمامی روش های دیالیزی که در بخش دیالیز انجام می شوند، می باشد.
4. **Patients Activation Measure(فعال سازی بیماران):** در این مطالعه منظور از فعال سازی بیماران میانگین نمره حاصل از پرسشنامه فعال سازی بیماران(PAM) که توسط هیبارد و همکاران توسعه یافته می باشد.
5. **Patients Treatment Adherence (تبعیت درمانی بیماران):** مقصود از تبعیت درمانی بیماران در این مطالعه میانگین نمره حاصل از مقیاس پایبندی به درمان بیماران مرحله نهایی نارسایی کلیوی (ESRD-AQ) می باشد.
6. **Weight Changes Patients (تغییرات وزنی بیماران):** در این مطالعه تغییر وزن بین‌دیالیزی (IDWG) با سنجش وزن پیش از اتصال به دستگاه و بلافاصله پس از پایان هر جلسه همودیالیز با رعایت مجموعه‌ای از استانداردها صورت خواهدگرفت؛ تمامی وزن‌کشی‌ها توسط یک ترازو دیجیتال پزشکی کالیبره‌شده (با کالیبراسیون ماهانه) انجام می شود، بیماران همواره با پوشیدن یک لباس سبک استاندارد دیالیز وزن می شوند و پیش از آن مثانه خود را خالی کرده، از مصرف غذا یا نوشیدنی پرهیز می نمایند. علاوه بر این، همه وسایل شخصی (کفش، کیف، جواهرات) پیش از سنجش وزن جدا می گردد و پرسنل آموزش‌دیده یا محقق کلیه مراحل را در زمان‌های ثابت (قبل از شروع و پس از اتمام دیالیز) با دقت به ثبت می‌رسانند تا از سازگاری داده‌ها و مقایسه منسجم تغییرات وزنی در طول دوره درمان اطمینان حاصل شود.

**2 -7- بررسي متون** **(Literature review):** (درصورت لزوم از صفحات اضافي استفاده و پيوست نماييد.)

1. Lightfoot و همکاران در سال ۲۰۲۴ یک مداخله سلامت دیجیتال با عنوان "کلیه‌های من و من" (My Kidneys & Me) را طراحی و تأثیر آن را در قالب یک کارآزمایی تصادفی‌شده با نام SMILE-K بررسی کردند. این مداخله با هدف ارائه آموزش‌های تخصصی در زمینه سلامت و سبک زندگی به بیماران مبتلا به بیماری مزمن کلیه[[1]](#footnote-2) (CKD) طراحی شده بود. در این مطالعه ۴۲۰ فرد بزرگسال با CKD مراحل ۳ و ۴ شرکت داشتند که به‌صورت تصادفی با نسبت ۲ به ۱ در دو گروه مداخله (n=280) و کنترل (n=140) تخصیص یافتند. پیامدها از جمله "میزان فعال‌سازی بیمار" (PAM-13) در آغاز مطالعه و پس از ۲۰ هفته اندازه‌گیری شد و تحلیل‌ها به دو صورت "بر اساس موارد کامل[[2]](#footnote-3)" (CC) و "بر اساس پروتکل[[3]](#footnote-4)" (PP) انجام گرفت. از میان شرکت‌کنندگان، ۲۱۰ نفر (۷۵٪) بیش از یک‌بار از پلتفرم MK&M استفاده کردند. یافته‌ها نشان داد که نمره PAM-13[[4]](#footnote-5) در گروه مداخله در مقایسه با گروه کنترل در پایان هفته بیستم افزایش یافته است (: CC به‌میزان ۳٫۱ واحد، (۰٫۲-۴/۶) ۹۵٪ CI:، P=0.065؛ :PP به‌میزان ۳٫۶ واحد،(۲/۰-۷) ۹۵٪ CI: ، P=0.041)، هرچند این افزایش در تحلیل CC از نظر آماری معنادار نبود. در میان افرادی که در آغاز مطالعه دارای سطح پایین فعال‌سازی بودند، تفاوت معنادار بین‌گروهی مشاهده شد که به نفع گروه MK&M بود (:CC به‌میزان ۶٫۶ واحد، ۹۵٪ CI: ۱٫۳ تا ۱۱٫۹، P=0.016؛ PP: به‌میزان ۹٫۲ واحد، ۹۵٪ CI: ۴٫۰ تا ۱۴٫۶، P<0.001). به‌طور کلی، استفاده از مداخله MK&M موجب بهبود فعال‌سازی بیماران نسبت به مراقبت استاندارد شد، اگرچه این اثر در کل نمونه از نظر آماری معنادار نبود و بیشترین سودمندی در میان بیماران با سطح پایین فعال‌سازی مشاهده گردید(36).
2. Nadri و همکاران در سال ۲۰۲۰ برنامه آموزشی ویژه‌ای را برای بیماران تحت همودیالیز مزمن طراحی و اثر آن را بر پایبندی این بیماران به محدودیت‌های غذایی و مایعات مورد بررسی قرار دادند. در این مطالعه مداخله‌ای که به‌صورت تک‌مرکزی انجام شد، ۵۰ بیمار در دو گروه ۲۵ نفره تخصیص یافتند؛ گروه کنترل بدون دریافت آموزش و گروه مداخله تحت پوشش برنامه آموزشی قرار گرفت. داده‌ها از طریق پرسشنامه ویژگی‌های جمعیت‌شناختی، پرسشنامه عدم پایبندی به رژیم غذایی و مایعات (DDFQ[[5]](#footnote-6)) و مقیاس کنترل مایعات در بیماران همودیالیز ([[6]](#footnote-7)FCHPS) جمع‌آوری شد. نتایج نشان داد که پس از اجرای برنامه آموزشی، میانگین افزایش وزن بین دیالیزی (کیلوگرم)، حجم اولترافیلتراسیون و فشار خون دیاستولیک در گروه مداخله به‌طور معنی‌داری کمتر از گروه کنترل بود. علاوه بر این، امتیازهای مربوط به چهار مولفه پرسشنامه DDFQ—شامل فراوانی و شدت عدم پایبندی به رژیم غذایی و فراوانی و شدت عدم پایبندی به محدودیت مایعات— در گروه مداخله به‌طور معناداری بهتر از گروه کنترل گزارش شد. در مجموع، این مطالعه نشان داد که اجرای یک برنامه آموزشی ساختارمند می‌تواند بهبود قابل‌توجهی در پایبندی بیماران همودیالیزی به رژیم غذایی و محدودیت مایعات و متعاقب آن تعییرات وزنی ایجاد کند(37).
3. Zhianfar و همکاران در سال ۲۰۲۰ یک مداخله چندوجهی را با هدف بهبود پایبندی به رژیم درمانی و کیفیت زندگی در بیماران دیالیزی اواخر مرحله ([[7]](#footnote-8)ESRD) اجرا نمودند. این کارآزمایی کنترل‌شده تصادفی در دو بخش همودیالیز بیمارستان شهروند ساری، مرکز استان مازندران، انجام شد؛ ۷۰ بیمار تحت دیالیز سرپایی پس از احتساب ۱۰٪ افت، به‌صورت تصادفی در گروه مداخله و کنترل جای گرفتند. مداخله شامل پخش ویدئوهای آموزشی مرتبط، برگزاری هشت جلسه گروهی رفتاردرمانی شناختی و پشتیبانی همتا از طریق تماس تلفنی بود. داده‌ها با استفاده از پرسشنامه‌های جمعیت‌شناختی، پرسشنامه اختلال خلقی بک ([[8]](#footnote-9)BDI-SF)، مقیاس حمایت اجتماعی چندبعدی (MSPSS)، [[9]](#footnote-10)پرسشنامه رضایت از کیفیت مراقبت پرستاری (PSNCQQ)[[10]](#footnote-11)، پرسشنامه پایبندی بیماران ESRD (ESRD-AQ)[[11]](#footnote-12) و مقیاس کیفیت زندگی سازمان جهانی بهداشت[[12]](#footnote-13) (WHOQOL-SF) در آغاز مطالعه و یک و سه ماه پس از مداخله جمع‌آوری شد. نتایج نشان از کاهش معنادار علائم افسردگی خودگزارش‌شده (P=0.001)، افزایش حمایت اجتماعی (P=0.001)، رضایت از مراقبت پرستاری (P=0.001)، کیفیت زندگی (P=0.001) و کاهش افزایش وزن بین دیالیزی[[13]](#footnote-14) (IDWG) (P=0.001) در گروه مداخله نسبت به خطوط پایه داشت. بالاترین رشد در امتیازهای ESRD-AQ چهارچوبی یک ماه پس از آغاز مداخله با میانگین تغییر ۱۳۱.۸۸ واحد مشاهده شد و تمامی زیرمقیاس‌های این پرسشنامه نیز تغییرات معنادار آماری را نشان دادند. این مطالعه نشان داد که راه‌اندازی یک مداخله کم‌هزینه و قابل اجرا بدون نیاز به منابع لجستیکی یا مالی عمده در سیستم‌های بهداشتی موجود، به‌ویژه در محیط‌های دارای منابع محدود، امکان‌پذیر است و می‌تواند چارچوبی علمی برای مداخلات مبتنی بر شواهد در ارائه مراقبت‌های بهداشتی فراهم آورد(38).
4. Zhang و همکاران در سال ۲۰۲۵ با هدف بررسی اثربخشی مداخلات سلامت دیجیتال بر بهبود پایبندی درمانی بیماران دیالیزی، یک متا-آنالیز سیستماتیک کارآزمایی‌های تصادفی‌شده را تا آوریل ۲۰۲۴ انجام دادند. در این مطالعه ۱۷ کارآزمایی با ۱۴۳۸ بیمار دیالیزی مورد بررسی قرار گرفت و میانگین تفاوت‌های استانداردشده[[14]](#footnote-15) (SMD) و بازه‌های اطمینان ۹۵٪ برای شاخص‌های پایبندی محاسبه شد. کیفیت شواهد به کمک روش GRADE ارزیابی گردید. نتایج نشان داد که مداخلات سلامت دیجیتال بهبود قابل‌توجهی در پایبندی کلی درمان ایجاد می‌کنند (SMD=1.88؛ ۹۵٪ CI: 0.46–3.29؛ چهار مطالعه؛ شواهد با اطمینان پایین). به‌طور خاص، در پایبندی به مصرف دارو (SMD=1.45؛ ۹۵٪ CI: 0.38–2.52؛ چهار مطالعه، ۳۰۰ بیمار؛ اطمینان پایین) و پایبندی به جلسات دیالیز (SMD=1.88؛ ۹۵٪ CI: 0.46–3.29؛ چهار مطالعه، ۲۴۵ بیمار؛ اطمینان پایین) بهبودهای بزرگ مشاهده شد. همچنین بهبودهای متوسط در پایبندی به رژیم غذایی (SMD=0.58؛ ۹۵٪ CI: 0.25–0.91؛ چهار مطالعه، ۳۴۴ بیمار؛ اطمینان متوسط) و مدیریت مایعات (SMD=−0.36؛ ۹۵٪ CI: −0.64 تا −0.07؛ هفت مطالعه، ۶۱۹ بیمار؛ اطمینان متوسط) گزارش گردید. به‌طور کلی، این متاآنالیز نشان داد که مداخلات سلامت دیجیتال به طور مؤثری ابعاد مختلف پایبندی درمانی بیماران دیالیزی را بهبود می‌بخشد و ارزش ادغام آن‌ها در عمل بالینی روزمره را برجسته می‌سازد(39).
5. Pour و همکاران در سال ۲۰۲۰ یک کارآزمایی بالینی تک‌کور پیش‌آزمون–پس‌آزمون تصادفی‌شده را در بین ۶۳ بیمار مبتلا به پرفشاری خون بزرگسال در یک بیمارستان نظامی تهران اجرا کردند تا اثربخشی آموزش تعاملی و غیرتعاملی از طریق پیامک کوتاه[[15]](#footnote-16) (SMS) را بر پایبندی به درمان و فشار خون ارزیابی کنند. در این مطالعه، تمامی بیماران ابتدا در یک جلسه ۴۵ دقیقه‌ای آموزش‌های پایه درباره [[16]](#footnote-17)HTN و پایبندی به درمان دریافت کردند و سپس گروه‌های SMS تعاملی[[17]](#footnote-18) و غیرتعاملی برای چهار ماه، چهار پیام هفتگی دریافت نمودند؛ گروه کنترل تنها آموزش اولیه را داشت. پایبندی درمان با «پرسشنامه پایبندی بیماران پرفشاری خون[[18]](#footnote-19)» و فشار خون سیستولیک و دیاستولیک در آغاز و ماهانه اندازه‌گیری شد. نتایج نشان داد که امتیاز پایبندی در گروه SMS تعاملی به‌طور معناداری از ۸۱٫۴۳±۹٫۱۵ به ۸۹٫۶۷±۴٫۴۷ افزایش یافت (P=0.003)، در حالی که تغییرات گروه SMS غیرتعاملی (۸۳٫۲۴±۷٫۱۸؛ P=0.15) و کنترل (۸۷٫۸۶±۶٫۶۲؛ P=0.16) معنادار نبود و تفاوت میان گروه‌ها پس از مداخله نیز معنادار بود (P=0.004). اگرچه فشار خون سیستولیک و دیاستولیک در دو گروه مداخله کاهش معنی‌داری داشت (P<0.05)، اما تغییرات فشار خون بین گروه‌ها در چهار اندازه‌گیری زمانی تفاوت آماری معناداری نشان نداد (P>0.05). بدین‌ترتیب، آموزش تعاملی مبتنی بر SMS در بهبود پایبندی به درمان مؤثر ارزیابی شد، ولی هیچ‌یک از دو روش پیامکی نتوانست فشار خون را به‌طور قابل‌توجه کاهش دهد(40).

**1-8- اهداف و فرضيات پژوهش (Objectives & Hypotheses):**

**2-8-1- هدف کلی** (General objectives)**:**

تاثیر و مقایسه روش آموزشی مبتنی بر ویدیوی تعاملی با روش آموزشی مبتنی بر ویدیوی معمولی بر فعال‌سازی، تبعیت درمانی و تغییرات وزنی در بیماران دیالیزی

**2-8-2- اهداف اختصاصی** objectives) (Specific:

1. تعیین تاثیر آموزش مبتنی بر ویدیوی تعاملی بر فعال‌سازی بیماران دیالیزی تحت همودیالیز در بخش های همودیالیز بیمارستان‌های منتخب وابسته به دانشگاه علوم پزشکی شهید بهشتی
2. تعیین تاثیر آموزش مبتنی بر ویدیوی تعاملی بر تبعیت درمانی بیماران دیالیزی تحت همودیالیز در بخش های همودیالیز بیمارستان‌های منتخب وابسته به دانشگاه علوم پزشکی شهید بهشتی
3. تعیین تاثیر آموزش مبتنی بر ویدیوی تعاملی بر تغییرات وزنی در بیماران دیالیزی تحت همودیالیز در بخش های همودیالیز بیمارستان‌های منتخب وابسته به دانشگاه علوم پزشکی شهید بهشتی
4. تعیین تاثیر آموزش مبتنی بر ویدیوی معمولی بر فعال‌سازی بیماران دیالیزی تحت همودیالیز در بخش های همودیالیز بیمارستان‌های منتخب وابسته به دانشگاه علوم پزشکی شهید بهشتی
5. تعیین تاثیر آموزش مبتنی بر ویدیوی معمولی بر تبعیت درمانی بیماران دیالیزی تحت همودیالیز در بخش های همودیالیز بیمارستان‌های منتخب وابسته به دانشگاه علوم پزشکی شهید بهشتی
6. تعیین تاثیر آموزش مبتنی بر ویدیوی معمولی بر تغییرات وزنی در بیماران دیالیزی تحت همودیالیز در بخش های همودیالیز بیمارستان‌های منتخب وابسته به دانشگاه علوم پزشکی شهید بهشتی
7. مقایسه روش آموزشی مبتنی بر ویدیوی تعاملی با روش آموزشی مبتنی بر ویدیوی معمولی بر فعال‌سازی، تبعیت درمانی و تغییرات وزنی در بیماران دیالیزی

**2-8-3- اهداف كاربردي** (Applied objectives)**:**

**2-8-4- فرضيات / سؤالات** (Hypotheses / Questions):

1. آموزش مبتنی بر ویدیوی تعاملی بر فعال‌سازی بیماران دیالیزی تحت همودیالیز در بخش های همودیالیز بیمارستان‌های منتخب وابسته به دانشگاه علوم پزشکی شهید بهشتی موثر است.
2. آموزش مبتنی بر ویدیوی تعاملی بر تبعیت درمانی بیماران دیالیزی تحت همودیالیز در بخش های همودیالیز بیمارستان‌های منتخب وابسته به دانشگاه علوم پزشکی شهید بهشتی موثر است.
3. آموزش مبتنی بر ویدیوی تعاملی بر تغییرات وزنی بیماران دیالیزی تحت همودیالیز در بخش های همودیالیز بیمارستان‌های منتخب وابسته به دانشگاه علوم پزشکی شهید بهشتی موثر است.
4. آموزش مبتنی بر ویدیوی معمولی بر فعال‌سازی بیماران دیالیزی تحت همودیالیز در بخش های همودیالیز بیمارستان‌های منتخب وابسته به دانشگاه علوم پزشکی شهید بهشتی موثر است.
5. آموزش مبتنی بر ویدیوی معمولی بر تبعیت درمانی بیماران دیالیزی تحت همودیالیز در بخش های همودیالیز بیمارستان‌های منتخب وابسته به دانشگاه علوم پزشکی شهید بهشتی موثر است.
6. آموزش مبتنی بر ویدیوی معمولی بر تغییرات وزنی بیماران دیالیزی تحت همودیالیز در بخش های همودیالیز بیمارستان‌های منتخب وابسته به دانشگاه علوم پزشکی شهید بهشتی موثر است.
7. اثربخشی آموزش مبتنی بر ویدیوی تعاملی با آموزش مبتنی بر ویدیوی معمولی بر فعال‌سازی، تبعیت درمانی و تغییرات وزنی بیماران دیالیزی قابل مقایسه می باشد.

**2-9- روش و تکنِيک ونحوهاجرای مطالعه (Method of Research):**

**2-9-1- طراحي و روش اجراي پروژه** ((Study design & Method **:**

**13- روش پژوهش(نوع مطالعه، مختصری از روش کار و ابزار پژوهش نوشته شود):**

این مطالعه یک کارآزمایی تصادفی کنترل شده با سه گروه موازی می باشد. نمونه پژوهش را بیماران تحت همودیالیز در بخش های همودیالیز بیمارستان‌های منتخب وابسته به دانشگاه علوم پزشکی شهید بهشتی تشکیل خواهند داد. معیارهای ورود به مطالعه شامل موارد زیر خواهد بود: (الف) تمایل به شرکت در مطالعه، (ب) داشتن سواد خواندن و نوشتن، (ج) داشتن هوشیاری و آگاه بودن به زمان، مکان و شخص برای پاسخ به سؤالات، (د) نداشتن سابقه اختلالات شنوایی و بینایی، (ه) نداشتن اختلال شناختی، (و) داشتن تلفن همراه شخصی یا هر ابزار دیگری که بتوان ویدیو تعاملی را اجرا کرد و توانایی استفاده از آن، (ز) عدم استفاده از داروهای روانگردان، (ح) تشخیص دقیق بیماری مزمن کلیه که توسط متخصص نفرولوژی تأیید شده باشد و داشتن پرونده پزشکی در بخش دیالیز و (خ) گروه سنی ۱۸ تا ۶۵ سال. معیارهای خروج از مطالعه شامل (الف) انصراف از مطالعه در هر مرحله، (ب) عدم دریافت و تماشای ویدیو های ارائه شده، (ج) فوت بیمار، و (د) انتقال به مرکز درمانی خارج از مراکز تحت پوشش دانشگاه علوم پزشکی شهید بهشتی خواهد بود. پس از تأیید مطالعه توسط کمیته اخلاق، محققین با اخذ مجوزهای مربوطه، مطالعه را آغاز خواهندکرد. در ابتدا، یک مرور متون گسترده جهت دستیابی به بهینه ترین محتوای آموزشی صورت خواهد گرفت. این فرآیند شامل استخراج مقالات، کتاب‌ها، برنامه‌ها و دستورالعمل های بالینی و آموزشی مرتبط خواهد بود. در این زمینه پایگاه های اطلاعاتی انگلیسی زبان ار جمله PubMed, Scopus, Web of Science, Embase مورد جستجوی سیستماتیک قرار خواهند گرفت. از بین پایگاه های فارسی زبان نیز SID, Magiran, Iranmedex مورد جستجو قرار خواهند گرفت. جستجو ها با استفاده از کلیدواژه های “Patient Activation,” “Treatment Adherence,” “Hemodialysis” و مترادف ها و واژگان جایگزین صورت خواهد گرفت. بازه زمانی جستجوها از ابتدای هر پایگاه تا ژوئن 2025 خواهد بود. سپس، محتوای اولیه برنامه توسعه خواهد یافت و سپس ارزیابی خواهد شد. پنج نفر از پرستاران بخش مراقبت ویژه با تکمیل چک‌لیستی، جامعیت برنامه را ارزیابی و نقاط نیازمند آموزش بیشتر را مشخص خواهند کرد که بازخوردهای ایشان در نسخه نهایی برنامه لحاظ خواهد شد. پس از تکمیل برنامه آموزشی، محتوای آن توسط ده تن از اعضای هیئت‌علمی و متخصصان بیهوشی اعتبارسنجی خواهد شد. بر اساس ارزیابی کارشناسان، مطالب تکمیلی تولید و اصلاحات لازم صورت خواهد پذیرفت. پس از آماده شدن محتوای آموزشی، سه بیمارستان از بین بیمارستان های دانشگاه علوم پزشکی شهید بهشتی به صورت تصادفی انتخاب خواهند شد. سپس برای تخصیص نوع مداخله به مراکز از نه پاکت که هر کدام نام یک بیمارستان و یکی از گروه‌های A، B یا C روی آن درج شده، سه پاکت به‌صورت تصادفی انتخاب خواهند شد تا نوع مداخله هر بیمارستان مشخص شود. شرکت‌کنندگان نیز بر اساس محل دریافت درمان به گروه‌های A (ویدیو تعاملی)، B (ویدیو معمولی) و C (گروه کنترل) اختصاص خواهند یافت. سپس در داخل هر بیمارستان بیماران با استفاده از جدول اعداد تصادفی از میان تمام بیماران تحت همودیالیز انتخاب خواهند شد. برای اطمینان از همسانی گروه‌ها، قبل از مداخله داده‌های جمعیت‌شناختی پایه و متغیر های اصلی مطالعه از هر سه گروه گردآوری خواهد شد. سپس گروه A ویدیو تعاملی را دریافت خواهد کرد. این برنامه شامل ده جلسه آموزشی 30 دقیقه ای خواهد بود. روش انتقال محتوا شامل سخنرانی و پرسش و پاسخ می با‌شد. گروه B ویدیو معمول را دریافت خواهند کرد؛ این دوره مشتمل بر ده ویدئوی ۳۰ دقیقه‌ای خواهد بود که طی یک ده روز ارائه خواهد شد. گروه کنترل (C) تنها آموزش‌های روتین را دریافت خواهد کرد. بلافاصه پس از اتمام جلسات آموزشی، یک و سه ماه پس از آن پیامدهای مورد نظر ارزیابی خواهند شد. بعد از پایان ارزیابی ها جهت رعایت اصول اخلاقی تمامی محتواهای ارائه شده به هر سه گروه داده خواهد شد.

**روش آموزش برای گروه مداخله با ویدیوی تعاملی:**

این مداخله آموزشی شامل دو مرحله و در مجموع مدت زمان شش ساعت اجرا خواهد شد. بیماران در قالب گروه‌های 5 نفره و تحت هدایت یک مدرس با تجربه و آشنا به سیستم آموزش ویدیویی تعاملی در این مطالعه شرکت خواهند کرد.

**مرحله اول – آموزش مبتنی بر ویدیوی تعاملی (10 جلسه 30 دقیقه ای):**

بیماران از طریق وب‌سایت https://spotplayer.ir/ وارد سامانه شده و ویدیوی تعاملی مرتبط با بیماری کلیوی مرحله آخر و درمان های جایگزین به طور مفصل مشاهده خواهند کرد. این ویدیو شامل ده جلسه 30 دقیقه ای بوده که به صورت یک جلسه در روز برای بیماران بارگذاری می شود. در حین مشاهده، سؤالاتی به صورت پاپ‌آپ ظاهر می شوند که بیماران باید به آن‌ها پاسخ دهند. انتخاب‌های متفاوت منجر به نمایش سناریوهای آموزشی متفاوت خواهد شد. همچنین، در بخش‌هایی از ویدیو، پرسش‌های چندگزینه‌ای درباره مفاهیم مرتبط ارائه خواهد شد که بیماران باید پاسخ‌های خود را در سیستم ثبت کنند و مدرس امکان مشاهده پاسخ‌ها را در پنل مدیریتی خواهد داشت. لازم به ذکر است تمامی بیماران از قبل آموزش های لازم جهت استفاده از ویدیو های تعاملی را دیده و پرسش و پاسخ های لازم جهت رسیدن به مهارت بالا در خصوص استفاده از ویدیو تعاملی انجام خواهد شد.

**مرحله دوم – جمع‌بندی و تثبیت آموزش ها (۱ ساعت):**

پس از اتمام ده جلسه ویدیوی تعاملی، بیماران در قالب گروهی به بحث و تحلیل سناریوها و مطالب و سؤالات مطرح‌شده خواهند پرداخت. مدرس با هدایت بحث‌ها، نتایج را جمع‌بندی کرده و عملکرد کلی گروه‌ها را ارزیابی خواهد کرد. در این مرحله، با هدایت مدرس، بیماران به بررسی ابعاد پیچیده بیماری پرداخته و اطلاعات کلیدی را استخراج خواهند کرد. در پایان، مدرس با ارائه توضیحات نظری هدفمند، تحلیل‌های بیماران را تکمیل و تقویت خواهد کرد.

**روش آموزش برای گروه مداخله با ویدیوی معمولی:**

این مداخله آموزشی شامل دو مرحله و در مدت زمان شش ساعت اجرا خواهد شد. بیماران در قالب گروه‌های 5 نفره و تحت هدایت یک مدرس با تجربه و آشنا به سیستم آموزش ویدیویی تعاملی در این مطالعه شرکت خواهند کرد.

**مرحله اول – آموزش مبتنی بر ویدیوی تعاملی (10 جلسه 30 دقیقه ای):**

بیماران از طریق وب‌سایت https://spotplayer.ir/ وارد سامانه شده و ویدیوهای مرتبط با بیماری کلیوی مرحله آخر و درمان های جایگزین به طور مفصل را مشاهده خواهند کرد. این ویدیو شامل ده جلسه 30 دقیقه ای بوده که به صورت یک جلسه در روز برای بیماران بارگذاری می شود.

**مرحله دوم – جمع‌بندی و تثبیت آموزش ها (۱ ساعت):**

پس از اتمام ده جلسه ویدیویی، بیماران در قالب گروهی به بحث و تحلیل سناریوها و مطالب و سؤالات مطرح‌شده خواهند پرداخت. مدرس با هدایت بحث‌ها، نتایج را جمع‌بندی کرده و عملکرد کلی گروه‌ها را ارزیابی خواهد کرد. در این مرحله، با هدایت مدرس، بیماران به بررسی ابعاد پیچیده بیماری پرداخته و اطلاعات کلیدی را استخراج خواهند کرد. در پایان، مدرس با ارائه توضیحات نظری هدفمند، تحلیل‌های بیماران را تکمیل و تقویت خواهد کرد.

**گروه کنترل:**

این گروه همان آموزش های روتین هر مرکز که شامل آموزش به بیمار توسط پرستاران، پمفلت آموزشی و پیگیری های آموزشی توسط پرستار پیگیر بخش خواهد بود.

**2-9-2- نوع مطالعه** ((Type of study**:**

کارآزمایی بالینی سه گروهی

**2_9_3روش جمع آوری اطلاعات:**

از طریق پرسشنامه های مجزا

**2-9-4- ابزار و روش جمع‌آوري اطلاعات** ((Data collection techniques**:**

1. **پرسشنامه اطلاعات جمعیت شناختی:** این پرسشنامه شامل دو بخش می باشد:

بخش اول داده‌های جمعیت‌شناختی شامل سن، جنس، وضعیت تحصیلات، وضعیت تاهل، مدت زمان تشخیص بیماری، مدت زمان از شروع اولین دیالیز، وضعیت شغلی، محل سکونت و بیماری های همراه می باشد.

بخش دوم داده های بالینی شامل میزان مصرف نمک در روز (به گرم)، عوارض و غیره، و میزان ادرار (به میلی‌لیتر در روز)، تعداد جلسات همودیالیز در هفته، میزان مصرف آب که از پرونده‌های پزشکی شرکت‌کنندگان قبل از مداخله در ابتدا جمع‌آوری می شود.

1. **پرسشنامه فعال سازی بیمار :** مقیاس فعال‌سازی بیمار (PAM) که توسط هیبارد و همکاران توسعه یافته است، شامل ۱۳ سؤال بوده و ابزاری معتبر و قابل اتکا برای سنجش فعال‌سازی بیماران در حوزه نفرولوژی به‌شمار می‌آید. پاسخ‌ها در این مقیاس بر پایه یک مقیاس لیکرت پنج‌گزینه‌ای («کاملاً مخالفم» = ۱، «مخالفم» = ۲، «موافقم» = ۳، «کاملاً موافقم» = ۴ و «غیرقابل‌اعمال» = فاقد امتیاز) ثبت می‌شوند و مجموع نمرات خام در بازه ۱۳ تا ۵۲ قرار دارد؛ نمرات پایین‌تر نشان‌دهنده فعال‌سازی کمتر و نمرات بالاتر نمایانگر فعال‌سازی بیشتر است(41). مطابق دستورالعمل نمره‌گذاری ارائه‌شده توسط هیبارد و همکاران، نمره کلی فعال‌سازی به مقیاس ۰–۱۰۰ استاندارد شده و در چهار سطح تفکیک می‌گردد: سطح یک «بی‌تفاوت و دچار سردرگمی» (نمره ≤ ۴۷)، سطح دو «آگاه‌شدن در وظایف خودمدیریتی» (۴۷.۱–۵۵.۱)، سطح سه «اقدام‌ورزی» (۵۵.۲–۶۷) و سطح چهار «حفظ رفتارها و پیشرفت بیشتر» (> ۶۷.۱). مطالعات پیشین پایایی این مقیاس را با ضریب آلفای کرونباخ ۸۷٪ تأیید کرده‌اند(42). ضریب آلفای کرونباخ برای نسخه فارسی PAM، ۹۱٪ به دست آمد(20).
2. **پرسشنامه تبعیت درمانی :** مقیاس پایبندی به درمان بیماران مرحله نهایی نارسایی کلیوی (ESRD-AQ) یک ابزار خوداظهاری است که شامل ۴۶ سؤال در پنج بخش می‌شود و به‌منظور ارزیابی پایبندی بیمار به درمان در چهار بعد حضور در جلسات همودیالیز، مصرف دارو، محدودیت مایعات و رعایت توصیه‌های رژیمی طراحی شده است. بخش اول این پرسشنامه اطلاعات عمومی بیماران در مورد وضعیت ESRD و سابقه درمان جایگزین کلیه را می‌پرسد (۵ سؤال)، و چهار بخش بعدی به‌ترتیب شامل پایبندی به حضور در همودیالیز (۱۴ سؤال)، مصرف دارو (۹ سؤال)، محدودیت مایعات (۱۰ سؤال) و توصیه‌های رژیمی (۸ سؤال) می‌باشد. پاسخ‌ها در این ابزار به‌صورت ترکیبی از مقیاس لیکرت، سؤالات چندگزینه‌ای و فرمت «بله/خیر» ثبت می‌شود. مجموع امتیازات نهایی در بازه ۰ تا ۱۲۰۰ قرار دارد و امتیازات بالاتر نشانگر سطح بالاتر پایبندی به درمان است(43). پایایی این ابزار توسط رفیعی و همکاران (۲۰۱۴) با ضریب آلفای کرونباخ ۰٫۹۱ و ضریب پایایی بازآزمایی ۰٫۸۵ تأیید شد(44)؛ همچنین کیم و همکاران (۲۰۱۰) اعتبار محتوایی آن را با شاخص اعتبار محتوایی (CVI) برابر ۰٫۹۹ گزارش کردند(43).
3. **تغییرات وزنی بیماران:** در این مطالعه تغییر وزن بین‌دیالیزی (IDWG) با سنجش وزن پیش از اتصال به دستگاه و بلافاصله پس از پایان هر جلسه همودیالیز با رعایت مجموعه‌ای از استانداردها صورت خواهدگرفت؛ تمامی وزن‌کشی‌ها توسط یک ترازو دیجیتال پزشکی کالیبره‌شده (با کالیبراسیون ماهانه) انجام می شود، بیماران همواره با پوشیدن یک لباس سبک استاندارد دیالیز وزن می شوند و پیش از آن مثانه خود را خالی کرده، از مصرف غذا یا نوشیدنی پرهیز می نمایند. علاوه بر این، همه وسایل شخصی (کفش، کیف، جواهرات) پیش از سنجش وزن جدا می گردد و پرسنل آموزش‌دیده یا محقق کلیه مراحل را در زمان‌های ثابت (قبل از شروع و پس از اتمام دیالیز) با دقت به ثبت می‌رسانند تا از سازگاری داده‌ها و مقایسه منسجم تغییرات وزنی در طول دوره درمان اطمینان حاصل شود.

**2-9-5-جامعه مورد مطالعه**

بیماران دیالیزی مراجعه کننده به بخش های دیالیز بیمارستان های تحت پوشش دانشگاه علوم پزشکی شهید بهشتی

**2-9-6- روش نمونه گيري ومحاسبه اندازه نمونه** (Sampling method & Sample size calculation**):**

با توجه به نبود مطالعات پیشین که از نظر طراحی و مداخلات، شباهت نزدیکی با کارآزمایی حاضر داشته باشند، محاسبه حجم نمونه بر اساس داده‌های به‌دست‌آمده از یک مطالعه مقدماتی پایلوت انجام خواهد شد که توسط تیم تحقیقاتی اجرا می‌گردد. این مطالعه پایلوت، برآوردهایی از اندازه اثر و میزان تغییرپذیری فراهم خواهد کرد که برای محاسبه حجم نمونه مورد نیاز برای این کارآزمایی تصادفی کنترل‌شده سه‌گروهی با چهار زمان اندازه‌گیری، به کار گرفته می‌شود.

انتخاب این رویکرد اطمینان حاصل می‌کند که حجم نمونه دارای توان آماری کافی برای شناسایی تفاوت‌های بالینی معنادار بین گروه‌ها باشد، با در نظر گرفتن ویژگی‌های خاص جمعیت مورد مطالعه و مداخلات. بر اساس داده‌های پایلوت، اندازه اثر تخمین زده خواهد شد و با در نظر گرفتن سطح معنی‌داری ۰٫۰۵ (دوطرفه) و توان آماری ۹۵٪، برآورد کلی حجم نمونه به طور مساوی بین سه گروه مطالعه تقسیم خواهد شد.

با اتکا به داده‌های پایلوت در نبود منابع پیشین، طراحی مطالعه از دقت روش‌شناختی لازم برخوردار می‌شود و در عین حال، استفاده بهینه از منابع را تضمین کرده و توان آماری کافی برای پاسخ به اهداف اصلی تحقیق حفظ می‌گردد.

س

**2-9-5- روش‌هاي تجزيه و تحليل داده‌ها (**Data analyses) :

جهت تحلیل داده های کمی از آمار توصیفی مناسب شامل میانگین و انحراف معیار برای داده های با توزیع متقارن و میانه و دامنه میان چارکی برای توزیع های غیر متقارن و چوله استفاده خواهد شد. همچنین داده های کیفی با تعداد و درصد گزارش خواهند شد. همچنین از ضریب همبستگی پیرسن در صورت نرمال بودن متغیرها و یا درصورت نرمال نبودن توزیع ها، معادل ناپارامتری آن یعنی ضریب همبستگی اسپیرمن گزارش خواهد شد. برای تعدیل مخدوشگرها در بررسی ارتباط بین متغیر ها از مدلسازی خطی عمومی استفاده خواهد شد. همچنین ارتباط بین متغیرهای مد نظر با متغیرهای دموگرافیک کمی با ضریب همبستگی و متغیرهای دموگرافیک کیفی دو حالته با تی مستقل و کیفی چند حالته با آنالیز واریانس و در صورت لزوم، از معادل های ناپارامتری آنها بررسی خواهد شد. تحلیل ها با نرم افزار SPSS 26 خواهد شد. آزمونها در سطح معنی داری 5 درصد و به صورت دوطرفه خواهد بود.

**2-9-9- جدول متغيرها** (Variables table) :

| **روش اندازه گیری** | **واحد اندازه گیری** | **نوع متغیر** | | | | **نقش متغیر** | **نام متغیر** |
| --- | --- | --- | --- | --- | --- | --- | --- |
|  |  | **واحد اندازه گیری** | | | |  |  |
|  |  | **کمی** | | **کیفی** | |  |  |
|  |  | گسسته | پیوسته | رتبه ای | اسمی |  |  |
| پرسشنامه دموگرافیک | سال |  |  |  |  | زمینه ای | سن |
| پرسشنامه دموگرافیک | مرد، زن |  |  |  |  | زمینه ای | جنس |
| پرسشنامه دموگرافیک | متاهل، مجرد |  |  |  |  | زمینه ای | وضعیت تاهل |
| پرسشنامه دموگرافیک | شهر، روستا |  |  |  |  | زمینه ای | محل سکونت |
| پرسشنامه دموگرافیک | زیردیپلم، دیپلم، لیسانس و بالاتر |  |  |  |  | زمینه ای | تحصیلات |
| پرسشنامه دموگرافیک | ماه |  |  |  |  | زمینه ای | مدت زمان تشخیص بیماری |
| پرسشنامه دموگرافیک | ماه |  |  |  |  | زمینه ای | مدت زمان از شروع اولین دیالیز |
| پرسشنامه دموگرافیک | کارمند، بیکار، بازنشسته |  |  |  |  | زمینه ای | وضعیت شغلی |
| پرسشنامه دموگرافیک | دیابت، فشار خون، افسردگی، آنمی، نارسایی قلبی، سایر |  |  |  |  | زمینه ای | بیماری های همراه |
| پرونده های پزشکی | سندروم عدم تعادل، افت فشار خون، سایر |  |  |  |  | زمینه ای | عوارض |
| پرونده های پزشکی | تعداد |  |  |  |  | زمینه ای | تعداد جلسات همودیالیز در هفته |
| پرونده های پزشکی | میلی لیتر |  |  |  |  | زمینه ای | میزان مصرف آب |
| پرونده های پزشکی | گرم |  |  |  |  | زمینه ای | میزان مصرف نمک در روز |
| پرونده های پزشکی | میلی لیتر در روز |  |  |  |  | زمینه ای | میزان ادرار |
| سنجش وزن پیش از اتصال به دستگاه و بلافاصله پس از پایان هر جلسه همودیالیز | ترازو دیجیتال پزشکی کالیبره‌شده |  |  |  |  | وابسته | تغییرات وزنی بیماران |
| پرسشنامه فعال سازی بیمار | پرسشنامه استاندارد |  |  |  |  | وابسته | فعال سازی بیماران |
| مقیاس پایبندی به درمان بیماران مرحله نهایی نارسایی کلیوی | پرسشنامه استاندارد |  |  |  |  | وابسته | تبعیت درمانی |

**9-10-جدول مراحل اجراي پروژه** (GANTT) **:**

| **مراحل اجراي پروژه** | **زمان بر حسب ماه** | | | | | | | | | | | | | | | | | | | | | | | | **درصدپيشرفت كار** |
| --- | --- | --- | --- | --- | --- | --- | --- | --- | --- | --- | --- | --- | --- | --- | --- | --- | --- | --- | --- | --- | --- | --- | --- | --- | --- |
|  | **1** | **2** | **3** | **4** | **5** | **6** | **7** | **8** | **9** | **10** | **11** | **12** | **13** | **14** | **15** | **16** | **17** | **18** | **19** | **20** | **21** | **22** | **23** | **24** |  |
| جمع آوري اطلاعات، تكميل پرسشنامه و ورود به نرم افزار |  |  |  |  |  |  |  |  |  |  |  |  |  |  |  |  |  |  |  |  |  |  |  |  |  |
| تجزيه و تحليل اطلاعات |  |  |  |  |  |  |  |  |  |  |  |  |  |  |  |  |  |  |  |  |  |  |  |  |  |
| بازنگري و نتيجه گيري و نگارش متن |  |  |  |  |  |  |  |  |  |  |  |  |  |  |  |  |  |  |  |  |  |  |  |  |  |
|  |  |  |  |  |  |  |  |  |  |  |  |  |  |  |  |  |  |  |  |  |  |  |  |  |  |
|  |  |  |  |  |  |  |  |  |  |  |  |  |  |  |  |  |  |  |  |  |  |  |  |  |  |
|  |  |  |  |  |  |  |  |  |  |  |  |  |  |  |  |  |  |  |  |  |  |  |  |  |  |

**2-10- ملاحظات اخلاقي (Ethical considerations):**

ملاحظات اخلاقی که در این پژوهش مورد توجه قرار گرفت عبارتند از:

کد های اخلاقی مورد نظر شامل: کد شماره 1، 11، 12، 13، 14، 15، 16، 17، 19 ، 20 ، 25، ،27، 28، 29،30، 31

1- کسب اجازه نامه کتبی از ریاست محترم دانشکده پرستاری و مامایی دانشگاه علوم پزشکی شهید بهشتی.

2-کسب اجازه از معاونت پژوهشی دانشکده پرستاری ومعاونت پژوهشی دانشگاه علوم پزشکی شهید بهشتی.

3-ارائه معرفی نامه وکسب اجازه از مقامات مسئول

4-معرفی خود به واحدهای مورد پژوهش و تشریح اهداف و ماهیت پژوهش برای آنها و اخذ رضایت نامه کتبی از آنها.

5-توضیح به واحد های مورد پژوهش در ارتباط با اختیاری بودن شرکت در پژوهش و مختار بودن ادامه پژوهش و نیز محرمانه ماندن اطلاعات و عدم نیاز به ذکر نام در فرم مشخصات دموگرافیک.

6-ارائه خلاصه نتایج تحقیق به مسئولین بیمارستان های مورد مطالعه و کلیه افراد ذینفع در صورت درخواست.

**2 -11- محدوديت‌هاي پروژه، خطاهاي سيستماتيك احتمالي و راه‌هاي مقابله با آنها:**

**(Study limitations / Systematic errors and methods of control)**

در حال حاضر برای این مطالعه محدودیت خاصی وجود ندارد.

**2 -12-** **بودجه و هزينه‌ها (Budgeting & Expenses):**

**2-12-1-** هزينه پرسنلي با ذكر مشخصات كامل و ميزان اشتغال هر فرد و حق‌الزحمه آنها:

| رديف | نوع فعاليت | رتبه علمي | تعداد افراد | كل ساعات كار براي پروژه | حق‌الزحمه در ساعت | جمع |
| --- | --- | --- | --- | --- | --- | --- |
|  |  |  |  |  |  |  |
|  |  |  |  |  |  |  |
|  |  |  |  |  |  |  |
|  |  |  |  |  |  |  |
|  |  |  |  |  |  |  |
| **جمــــــع كـــــل** | | | | | |  |

**2-12-2-** هزينه آزمايشات و خدمات تخصصي كه توسط ديگر مؤسسات صورت مي‌گيرد:

| هزينه آزمايش با خدمات تخصصي | مركز سرويس دهنده | تعداد كل دفعات | هزينه براي هر دفعه | جمع (ريال) |
| --- | --- | --- | --- | --- |
|  |  |  |  |  |
|  |  |  |  |  |
|  |  |  |  |  |
|  |  |  |  |  |
|  |  |  |  |  |
| **جمــــــع كـــــل** | | | |  |

**2-12-3- فهرست وسايل و موادي كه بايد از اعتبار پروژه، داخل يا خارج كشور خريداري شود:**

***الف-*** مواد غيرمصرفي (سرمايه‌اي):

| نام دستگاه | كشور سازنده | شركت سازنده | آيا در ايران موجود است؟ | شركت فروشنده ايراني | تعداد لازم | قيمت واحد | قيمت كل |
| --- | --- | --- | --- | --- | --- | --- | --- |
|  |  |  |  |  |  |  |  |
|  |  |  |  |  |  |  |  |
|  |  |  |  |  |  |  |  |
|  |  |  |  |  |  |  |  |
| **جمــــــع كـــــل** | | | | | | |  |

***ب-*** مواد مصرفي:

| نام ماده | كشور سازنده | شركت سازنده | آيا در ايران موجود است؟ | شركت فروشنده ايراني | مقدار لازم | قيمت واحد | قيمت كل |
| --- | --- | --- | --- | --- | --- | --- | --- |
|  |  |  |  |  |  |  |  |
|  |  |  |  |  |  |  |  |
|  |  |  |  |  |  |  |  |
|  |  |  |  |  |  |  |  |
|  |  |  |  |  |  |  |  |
|  |  |  |  |  |  |  |  |
|  |  |  |  |  |  |  |  |
|  |  |  |  |  |  |  |  |
| **جمــــــع كـــــل** | | | | | | |  |

**2-12-4-** هزينه مسافرت (در صورت لزوم):

| مقصد | تعداد مسافرت در مدت اجراي پروژه و منظور آن | نوع وسيله نقليه | تعداد افراد | هزينه به ريال |
| --- | --- | --- | --- | --- |
|  |  |  |  |  |
|  |  |  |  |  |
|  |  |  |  |  |
|  |  |  |  |  |
| **جمــــــع كـــــل** | | | |  |

**2-12-5-** هزينه‌هاي متفرقه:

|  | **قيمت كل** |
| --- | --- |
| هزينه تكثير فرم‌ها و پرسش‌نامه‌هاي مورد نياز پروژه |  |
| هزينه تكثير نشريات و كتب مورد نياز پروژه |  |
| ساير هزينه‌ها (ذكر شود) ............. |  |
| **جمــــــع كـــــل** | |

**2-10-6- جمع هزينه‌هاي پروژه** (ريال):

| جمع هزينه‌هاي پرسنلي (2-10-1) |  |
| --- | --- |
| جمع هزينه‌هاي آزمايشات و خدمات تخصصي (2-10-2 ) |  |
| جمع هزينه‌هاي وسايل غيرمصرفي ( 2-10-3 )الف |  |
| جمع هزينه‌هاي مواد مصرفي (4-10-3) ب |  |
| جمع هزنيه‌هاي مسافرت (2-10-4 ) |  |
| جمع هزينه‌هاي متفرقه (2-10-5) |  |
| **جمـــع كــل** |  |

**2-12-7- مبلغ يا مبالغي كه از منابع ديگر كمك خواهد شد و نحوه مصرف آن:**

**بدين‌وسيله صحت مطالب مندرج فوق را كه در 14صفحه و 10 بند تنظيم و تكميل شده است تأييد مي‌نمايم.**

**نام ونام خانوادگي مجري (مجريان): تاريخ وامضا:**

**منابع( References) :**

1. Arad M, Goli R, Parizad N, Vahabzadeh D, Baghaei R. Do the patient education program and nurse-led telephone follow-up improve treatment adherence in hemodialysis patients? A randomized controlled trial. BMC Nephrology. 2021;22(1):119.

2. Lorenz EC, Kennedy CC, Rule AD, LeBrasseur NK, Kirkland JL, Hickson LJ. Frailty in CKD and transplantation. Kidney international reports. 2021;6(9):2270-80.

3. Itano S, Kanda E, Nagasu H, Nangaku M, Kashihara N. eGFR slope as a surrogate endpoint for clinical study in early stage of chronic kidney disease: from The Japan Chronic Kidney Disease Database. Clinical and Experimental Nephrology. 2023;27(10):847-56.

4. Ben Salah G, Farooqui M, Salem Alshammari M, Elghazali A, Kassem L, Ibrahim N, et al. Prevalence, types and disclosure of complementary and alternative medicine (CAM) use among chronic kidney disease (CKD) patients in Saudi Arabia. Journal of pharmaceutical policy and practice. 2023;16(1):89.

5. Alhamad MA, Almulhim MY, Alburayh AA, Alsaad RA, Alhajji AM, Alnajjar JS, et al. Factors affecting adherence to hemodialysis therapy among patients with end-stage renal disease attending in-center hemodialysis in Al-Ahsa Region, Saudi Arabia. Cureus. 2023;15(10):e46701.

6. Foy MD, Mang S, Mitchell A. End-stage renal disease and hemodialysis: Improving nursing care and patient experience. Nursing2024. 2023;53(8):46-51.

7. Dehghani A, Alishavandi S, Nourimajalan N, Fallahzadeh H, Rahmanian V. Prevalence of chronic kidney diseases and its determinants among Iranian adults: results of the first phase of Shahedieh cohort study. BMC Nephrology. 2022;23(1):203.

8. Clavé S, Tsimaratos M, Boucekine M, Ranchin B, Salomon R, Dunand O, et al. Quality of life in adolescents with chronic kidney disease who initiate haemodialysis treatment. BMC nephrology. 2019;20:1-10.

9. Hamrahian SM, Vilayet S, Herberth J, Fülöp T. Prevention of intradialytic hypotension in hemodialysis patients: Current challenges and future prospects. International Journal of Nephrology and Renovascular Disease. 2023:173-81.

10. Kim H, Jeong IS, Cho M-K. Effect of treatment adherence improvement program in hemodialysis patients: a systematic review and meta-analysis. International Journal of Environmental Research and Public Health. 2022;19(18):11657.

11. Kim H, Cho M-K. Factors influencing self-care behavior and treatment adherence in hemodialysis patients. International Journal of Environmental Research and Public Health. 2021;18(24):12934.

12. Saedi F, Dehghan M, Mohammadrafie N, Xu X, Hermis AH, Zakeri MA. Predictive role of spiritual health, resilience, and mental well-being in treatment adherence among hemodialysis patients. BMC nephrology. 2024;25(1):326.

13. Alilu L, Pazirofteh S, Habibzadeh H, Rasouli J. The impact of teach-back training method (TBTM) on treatment adherence in hemodialysis patients: a randomized controlled trial. Annals of Medicine and Surgery. 2024;86(5):2723-8.

14. Ma L-C, Liu Y-M, Lin Y-C, Liao C-T, Hung K-C, Chen R, et al. Factors influencing Self-management behaviors among hemodialysis patients. Journal of personalized medicine. 2022;12(11):1816.

15. Bossola M, Pepe G, Antocicco M, Severino A, Di Stasio E. Interdialytic weight gain and educational/cognitive, counseling/behavioral and psychological/affective interventions in patients on chronic hemodialysis: a systematic review and meta-analysis. Journal of Nephrology. 2022;35(8):1973-83.

16. Sajjadi SL, Ghafourifard M, Tayebi Khosroshahi H. A Randomized Controlled Clinical Trial of Individualized Patient Education on Hemodialysis Adequacy and Interdialytic Weight Gain. J Caring Sci. 2025;14(1):5-13.

17. Dammery G, Vitangcol K, Ansell J, Ellis LA, Smith CL, Carrigan A, et al. The Patient Activation Measure (PAM) and the pandemic: Predictors of patient activation among Australian health consumers during the COVID-19 pandemic. Health Expect. 2023;26(3):1107-17.

18. Kearns R, Harris-Roxas B, McDonald J, Song HJ, Dennis S, Harris M. Implementing the Patient Activation Measure (PAM) in clinical settings for patients with chronic conditions: a scoping review. Integrated Healthcare Journal. 2020;2(1):e000032.

19. Mei-Yu LIN, Wei-Shih W, Pham VAN, Pei-Shan T. Effects of patient activation intervention on chronic diseases: a meta-analysis. LWW; 2020. p. e116.

20. Mirmazhari R, Ghafourifard M, Sheikhalipour Z. Relationship between patient activation and self-efficacy among patients undergoing hemodialysis: a cross-sectional study. Renal Replacement Therapy. 2022;8(1):40.

21. Kang MS, Bae EJ. Effects of Food Craving, Patient Activation, and Family Support on Dietary Self-Care in Hemodialysis Patients. Journal of Korean Clinical Nursing Research. 2024;30(2):157-65.

22. Kwon YM, Hong SJ. The Effects of Self-Care Education Programs on Hemodialysis Patients. Journal of Korean Clinical Nursing Research. 2025;31(1):102-12.

23. Bossola M, Mariani I, Strizzi CT, Piccinni CP, Di Stasio E. How to Limit Interdialytic Weight Gain in Patients on Maintenance Hemodialysis: State of the Art and Perspectives. J Clin Med. 2025;14(6).

24. Bossola M, Mariani I, Sacco M, Antocicco M, Pepe G, Di Stasio E. Interdialytic weight gain and low dialysate sodium concentration in patients on chronic hemodialysis: a systematic review and meta-analysis. International Urology and Nephrology. 2024;56(7):2313-23.

25. Zanandreia M, Cattafesta M, Martins CA, Paixão MPCP, Soares FLP, Peterle FZ, et al. Socioeconomic, clinical and nutritional factors on interdialytic weight gain in haemodialysis users. Acta Paulista de Enfermagem. 2024;37:eAPE02062.

26. Fadlilah S, Nekada Y, Dede C, Lanni F, Saleha L, Lestiawati E, et al. Interdialytic Weight Gain (IDWG) and Complications of Intradialisis among Hemodialized Patients. International Medical Journal. 2021;28(6):620-4.

27. Kim YW, Choi GW, Chang SJ. The effect of cognitive behavioural therapy on interdialytic weight gain in hemodialysis patients: A systematic review and meta-analysis. Collegian. 2025.

28. Günen Yılmaz S, Yılmaz F. Evaluation of demographic and clinical risk factors for high interdialytic weight gain. Therapeutic Apheresis and Dialysis. 2022;26(3):613-23.

29. Pradhan R, Tripathy P, Kar D. Effect of multidisciplinary nursing intervention on interdialytic weight gain and quality of life among chronic hemodialysis patients: A randomized controlled trial. Journal of Integrative Nursing. 2025;7(1):12-20.

30. Düdener E, Hallaç S. The effect of the training of presence in care (TPinCare) on the care-oriented patient-nurse interaction and the caring behaviors of nurses working with oncology patients: A randomized controlled trial. Nurse Education in Practice. 2023;68:103566.

31. Clerkin R, Patton D, Moore Z, Nugent L, Avsar P, O'Connor T. What is the impact of video as a teaching method on achieving psychomotor skills in nursing? A systematic review and meta-analysis. Nurse Education Today. 2022;111:105280.

32. Liu Y, Chen J, Pan Y, Cai Y, Ge C, Chu H, et al. The effects of video based nursing education on perioperative anxiety and depression in patients with gastric cancer. Psychology, Health & Medicine. 2021;26(7):867-76.

33. Chao Y-C, Hu SH, Chiu H-Y, Huang P-H, Tsai H-T, Chuang Y-H. The effects of an immersive 3d interactive video program on improving student nurses' nursing skill competence: A randomized controlled trial study. Nurse education today. 2021;103:104979.

34. Natarajan J, Joseph MA, Al Shibli ZS, Al Hajji SS, Al Hanawi DK, Al Kharusi AN, et al. Effectiveness of an interactive educational video on knowledge, skill and satisfaction of nursing students. Sultan Qaboos University Medical Journal. 2022;22(4):546.

35. Voelker R. What Is Dialysis? JAMA. 2025;333(19):1752-.

36. Lightfoot CJ, Wilkinson TJ, Sohansoha GK, Gillies CL, Vadaszy N, Ford EC, et al. The effects of a digital health intervention on patient activation in chronic kidney disease. npj Digital Medicine. 2024;7(1):318.

37. Nadri A, Khanoussi A, Hssaine Y, Chettati M, Fadili W, Laouad I. Impact de l’éducation du patient en hémodialyse sur le respect des mesures diététiques et sur la restriction aux liquides. Néphrologie & Thérapeutique. 2020;16(6):353-8.

38. Zhianfar L, Nadrian H, Asghari Jafarabadi M, Espahbodi F, Shaghaghi A. Effectiveness of a multifaceted educational intervention to enhance therapeutic regimen adherence and quality of life amongst iranian hemodialysis patients: A randomized controlled trial (MEITRA study). Journal of Multidisciplinary Healthcare. 2020:361-72.

39. Zhang Z, Liang XT, He XW, Zhang X, Tang R, Fang RD, et al. Enhancing treatment adherence in dialysis patients through digital health interventions: a systematic review and meta-analysis of randomized controlled trials. Ren Fail. 2025;47(1):2482885.

40. Pour ER, Aliyari S, Farsi Z, Ghelich Y. Comparing the effects of interactive and noninteractive education using short message service on treatment adherence and blood pressure among patients with hypertension. Nursing and Midwifery Studies. 2020;9(2):68-76.

41. Nair D, Cavanaugh KL. Measuring patient activation as part of kidney disease policy: are we there yet? Journal of the American Society of Nephrology. 2020;31(7):1435-43.

42. Hibbard JH, Mahoney ER, Stockard J, Tusler M. Development and testing of a short form of the patient activation measure. Health services research. 2005;40(6p1):1918-30.

43. Kim Y, Evangelista LS, Phillips LR, Pavlish C, Kopple JD. The End-Stage Renal Disease Adherence Questionnaire (ESRD-AQ): testing the psychometric properties in patients receiving in-center hemodialysis. Nephrology nursing journal: journal of the American Nephrology Nurses' Association. 2010;37(4):377.

44. RAFIEE VL, Parvin N, MAHMOODI SG, Molaie E, Shariati A, Hasheminia S. Adherence to hemodialysis treatment and some related factors in hemodialysis patients admitted in Shahrekord Hajar hospital. 2014.

1. Chroic Kidney Disease [↑](#footnote-ref-2)
2. Complete Cases [↑](#footnote-ref-3)
3. Per-Protocol [↑](#footnote-ref-4)
4. Patient Activation Measure [↑](#footnote-ref-5)
5. dialysis diet and fluid non-adherence questionnaire [↑](#footnote-ref-6)
6. fluid control in hemodialysis patients scale [↑](#footnote-ref-7)
7. End-stage Renal Disease [↑](#footnote-ref-8)
8. Beck Mood Disorder Inventory [↑](#footnote-ref-9)
9. Multidimensional Social Support Scale [↑](#footnote-ref-10)
10. Patient Satisfaction with Nursing Care Quality Questionnaire [↑](#footnote-ref-11)
11. ESRD Patient Adherence Questionnaire [↑](#footnote-ref-12)
12. World Health Organization Quality of Life Scale [↑](#footnote-ref-13)
13. interdialysis weight gain [↑](#footnote-ref-14)
14. Standard Mean Differences [↑](#footnote-ref-15)
15. Short Message Service [↑](#footnote-ref-16)
16. Hypertension [↑](#footnote-ref-17)
17. Interactive [↑](#footnote-ref-18)
18. Hypertensive Patients Adherence Questionnaire [↑](#footnote-ref-19)
