# Supplementary material for: Comparing the effects of interactive and conventional video education on activation, treatment adherence, and weight changes in dialysis patients: A randomized clinical trial protocol
Source: PLoS One. 2025 Oct 15;20(10):e0334498. doi: 10.1371/journal.pone.0334498 (PMC12527215; doi:10.1371/journal.pone.0334498)
Supplement: Attachment — Submitted filename: Response to Reviewers.docx [file pone.0334498.s006.docx]

| **No.** | **Comment** | **Response and explanation** | **Location in Manuscript** |
| --- | --- | --- | --- |
| **Response to Required Revisions:**  1.Cluster randomisation with three hospitals: The protocol assigns one hospital to each arm. With only three clusters, intervention effects may be confounded by site-level differences and cannot be reliably separated from cluster effects. Please provide a strong justification for this design, at least give detailed information on comparability of these 3 centers, acknowledge it explicitly as a limitation, and describe how intra-cluster correlation will be addressed in both the pilot-based sample size calculation and subsequent analysis.  **Re:** We appreciate this insightful comment. Following the reviewers’ and editor’s recommendations, we have revised the study design. Instead of assigning entire hospitals to a single intervention arm, we now use individual-level randomization stratified by hospital and dialysis shift. This ensures balance across sites, prevents confounding by hospital-level characteristics, and eliminates the limitations associated with only three clusters.  2.Sample size and pilot study: Reviewers requested further detail on the planned pilot. Please specify how the pilot will be conducted (sample, measures, anticipated effect size estimates) and clarify how these data will inform the main trial’s power calculation. In addition, explain the rationale for using 95% power, which is higher than common practice, and justify feasibility of achieving this with the proposed design.  **Re:** Thank you for this important request for clarification. We have expanded the manuscript to describe the pilot study and to correct our power statement.  3.Instructor bias and workshop imbalance: Reviewer #1 raised valid concerns that the Phase 2 workshops may introduce instructor-driven bias and are not provided to the control arm. Please describe standardisation measures (e.g., facilitator scripts, checklists) to minimise bias, and justify why the control arm does not receive an equivalent structured session.  **Re:** We agree with the reviewer and have addressed this by using facilitator scripts, fidelity checklists, and standardized training to minimize instructor bias. Selected sessions will also be recorded for independent review. The control group receives the standard nurse-led education routinely provided in these centers; an additional structured workshop was not included to preserve the contrast with usual care, but all educational content will be offered to this group after outcome assessment.  4.Blinding of outcome assessment Reviewer #2 questioned why assessors are not blinded. Please provide justification for this choice and describe any measures you will use to minimise bias (e.g., independent assessors, scripted administration).  **Re:** We thank the reviewer for this point. While participants and facilitators cannot be blinded due to the nature of the interventions, outcome assessors will remain blinded to group allocation. They will administer questionnaires using standardized scripts and will extract clinical data independently. In addition, all analyses will be conducted on de-identified datasets labeled only as Group A, B, or C until the primary analyses are complete. These measures are intended to minimize detection and analytic bias.  5.Content validation: Clarify why ICU nurses and anaesthesia specialists were selected to review programme content rather than dialysis-experienced nurses or nephrologists. If possible, expand content validation to include specialists directly involved in haemodialysis care.  **Re:** Thank you for your instructive comment. This was a mistake and is now corrected.  6.Blinding of outcome assessment: Reviewer #2 questioned why assessors are not blinded. Please provide justification for this choice and describe any measures you will use to minimise bias (e.g., independent assessors, scripted administration).  **Re:** We thank the reviewer for this point. While participants and facilitators cannot be blinded due to the nature of the interventions, outcome assessors will remain blinded to group allocation. They will administer questionnaires using standardized scripts and will extract clinical data independently. In addition, all analyses will be conducted on de-identified datasets labeled only as Group A, B, or C until the primary analyses are complete. These measures are intended to minimize detection and analytic bias  7.Edits for clarity Replace all placeholders (e.g., “XXXX” for ethics approval and registration codes) with the correct identifiers. Revise Figure 3 to ensure baseline assessment is clearly shown to occur before intervention delivery.  **Re:** Thanks for your valuable comment. This is now corrected. | | | |
| 1 | For the recommended revisions, the authors should provide more detail on missing data handling (including assumptions for multiple imputation, number of imputations, and planned sensitivity analyses), clarify that interdialytic weight gain reflects both adherence and clinical factors and specify how potential confounders such as residual renal function and comorbidities will be addressed, ensure consistency of terminology by defining acronyms such as “PAM” at first use, review and streamline the introduction to focus on the specific evidence gap rather than extensive background epidemiology, and undertake minor grammatical edits to improve concision and clarity. The authors should also address the reviewers’ comments point by point in their rebuttal to ensure all issues raised have been adequately resolved. | We sincerely thank the editor and reviewer for these constructive suggestions, which have significantly improved the clarity and rigor of our manuscript. The following revisions have been made:  Missing data handling:  We have expanded the Statistical Analysis section to provide comprehensive details on missing data handling. Specifically, we now describe the assumptions for multiple imputation, the number of imputations, and the planned sensitivity analyses, including complete-case analysis, pattern-mixture models, and delta-adjustment approaches.  Interdialytic weight gain (IDWG):  We clarified that IDWG reflects not only fluid adherence but also clinical factors such as residual renal function and comorbidities.  Potential confounders:  We specified how residual renal function, comorbidities, and other relevant covariates will be recorded and incorporated into adjusted analyses to minimize potential confounding bias.  Consistency of terminology:  All acronyms are now defined at their first occurrence. For example, “Patient Activation Measure (PAM)” has been fully clarified at first use.  Introduction revision:  The Introduction has been streamlined to emphasize the evidence gap addressed by this trial and the novelty of our study, with less emphasis on broad epidemiological background.  Language and grammar:  Minor grammatical and stylistic edits have been undertaken throughout the manuscript to enhance concision, clarity, and readability. | Whole Manuscript |
|  |  |  | Whole Manuscript |
| **Reviewer 1:**  This study protocol addresses an important and timely question on how best to enhance patient activation and adherence in hemodialysis through interactive versus conventional video education. The design is rigorous, with a three-arm, cluster-randomized trial framework, but there are several methodological issues that need clarification to ensure validity and interpretability.  **Response:**  We sincerely thank you for taking the time to carefully read and evaluate our study protocol. We greatly appreciate your thoughtful and constructive comments and the attention you paid to the study’s importance, design, and methodological considerations. Your expertise and the time you invested in reviewing our manuscript are very valuable to us; they will help improve the clarity, rigor, and interpretability of the final version. We will take your observations into account in our revision and are grateful for your contribution to strengthening this work. | | | |
| 1. | How will you decrease potential instructor bias in the interactive video education arm in Phase 2, given that the instructor facilitates critical discussions, guides extraction of salient clinical concepts, and synthesizes group findings? This process may introduce intervention bias by directing participants to retain knowledge specific to the PAM questions. | To minimize potential instructor bias in Phase 2, we will implement several fidelity safeguards: (1) a facilitator manual with a scripted guide, learning objectives, and structured timelines; (2) a fidelity checklist completed after each session and verified by the study monitor; (3) standardized training and competency testing for all facilitators (≥85% pass threshold); and (4) sampled audio/video recordings independently reviewed by blinded external evaluators, with corrective feedback provided early in the study. These measures will ensure that observed effects reflect the interactive video components rather than facilitator influence. | 10 |
| 2. | Why is the 1-hour workshop implemented only in the interactive video and conventional video arms, while the usual care control arm does not receive a workshop? | We thank the reviewer for this important point. The control arm was intentionally defined as ‘usual care’ (nurse-led education, pamphlets, routine nurse follow-up) to preserve external validity and to compare each video modality against the standard practice patients currently receive. Adding a structured, instructor-led workshop to the control arm would convert the control into an active educational intervention and therefore preclude a clear comparison between the study interventions and real-world care. To address concerns about differential attention and facilitator bias, we have implemented the following: standardized facilitator manuals and competency certification for all instructors; fidelity checklists completed after every session; logging of participant contact time; and planned analytic strategies including ITT, per-protocol and as-treated analyses, mixed-effects models with cluster adjustment, and sensitivity analyses (including as-treated/CACE analyses) to evaluate the potential influence of differential contact. Finally, all participants in the control arm will be offered access to the video modules and workshop materials after final outcome collection to maintain ethical equipoise. | - |
| 3. | How will you ensure content equivalence across interventions, particularly in terms of detail and knowledge structure? | We appreciate the reviewer’s concern regarding content equivalence across the video interventions. To ensure that any observed differences are attributable solely to delivery modality rather than content, both the interactive and conventional videos were developed from a single master content blueprint. The blueprint specifies identical learning objectives, clinical concepts, scenarios, knowledge units, and sequencing of material. | 10, 16 |
| 4. | How will you address possible contamination between patients within the same dialysis facility or even the same dialysis session? | We appreciate the reviewer’s concern. As described in the manuscript, we have taken several steps to minimize and monitor contamination. Video content will be provided through secure, password-protected accounts, and workshops will be conducted separately for each group with dedicated facilitators. At follow-up visits, participants will be asked whether they were exposed to materials from other arms, and self-reports will be used to estimate cross-exposure. If contamination exceeds 20%, this will be clearly reported, and sensitivity analyses will be performed to assess the robustness of findings. | 15 |
| 5. | In Figure 3, should baseline assessment occur before the intervention? | Thanks for your valuable comment. This is now corrected. | Figure 3 |
| 6. | The abbreviation “PAM tool” is not mentioned before page 4, paragraph 2. | Thank you for pointing this out. We defined the abbreviation “PAM tool” at its first mention in the manuscript to ensure clarity for the reader. | 4 |
| 7. | Please provide the ethics committee code (XXXX) and ClinicalTrials.gov registration number (XXXX). | Thank you for this comment. We added this to manuscript. | 8 |
| 8. | Why will the program content be assessed by ICU nurses rather than hemodialysis nurses? | Thanks for your valuable comment. This is now corrected. | 10 |
| 9. | How were the ten faculty members who validated the program content selected? | Thanks, full description is now added. | 10 |
| 10. | Why were anesthesia specialists involved as key evaluators of the program content, rather than nephrologists or internists? | Thanks for your valuable comment. This is now corrected. | 10 |
| **Reviewer 2:**  The research team plans a clinical trial to assess the impact of interactive and conventional video-based education on patient activation, treatment adherence, and inter-dialytic weight gain in a dialysis population.  **Response:**  We sincerely thank you for taking the time to carefully review my manuscript and provide such valuable feedback. Your comments and suggestions were extremely helpful and greatly contributed to improving the quality of the work. | | | |
| 1. | Sample size will be determined based on a pilot study. However, there is a lack of pilot study information. | We thank the reviewer for this observation. In the revised manuscript, we have elaborated on the pilot study that will precede the definitive trial. The pilot will be conducted in the same dialysis units, with approximately 12 participants per arm for feasibility assessment, or up to 35 per arm if variance estimation is required. Its objectives include estimating parameters for sample size calculation, assessing recruitment and retention, evaluating adherence and fidelity, and identifying potential operational challenges. These details are now clearly described in the Sample Size section. | 7 |
| 2. | Please clarify why to use 95% power for the sample size calculation as it is much higher than the commonly used level. | We appreciate the reviewer’s attention to this point. We confirm that the intended statistical power for the definitive trial is 80%, which is the conventional level for clinical research. Any previous reference to 95% was unintentional and has been corrected in the revised manuscript. The sample size section now clearly specifies that calculations will be based on 80% power and a two-sided α of 0.05. | 7 |
| 3. | Please provide some more details on the missing data imputation | Thank you for this helpful suggestion. We have expanded the Statistical Analysis section to provide more details. Specifically, we state that missing data will be handled using multiple imputation by chained equations, with at least 20 imputations. Appropriate conditional models will be applied according to variable type (predictive mean matching for continuous variables, logistic or multinomial regression for categorical variables). Convergence of chains will be checked, and pooled estimates will be obtained using Rubin’s rules. Sensitivity analyses, including complete-case analysis and pattern-mixture/tipping-point approaches, will also be conducted to assess the robustness of the results. | 19 |
| 4. | Please clarify why outcome accessors will not be blinded as this may introduce the bias. | Thank you for your instructive comment. This was a mistake and is now corrected. | 14 |
| 5. | Please provide the justification for the feasibility to implement the study within four months as planned. | We appreciate the reviewer’s comment regarding the feasibility of completing the study within four months. We would like to clarify that the protocol does not specify a fixed overall study duration. The implementation of the interventions and follow-up assessments will be conducted according to patient availability during routine hemodialysis sessions and the capacity of the participating centers. Therefore, any reference to a four-month timeline reflects an estimated projection rather than a predetermined study period, and all study procedures will be executed flexibly in alignment with the operational schedule of the dialysis units and participant engagement. | 9 |
